# Bridging the membrane lipid divide: bacteria of the FCB group superphylum have the potential to synthesize archaeal ether lipids

**DOI:** 10.1038/s41396-020-00772-2

**Published:** 2020-09-14

**Authors:** Laura Villanueva, F. A. Bastiaan von Meijenfeldt, Alexander B. Westbye, Subhash Yadav, Ellen C. Hopmans, Bas E. Dutilh, Jaap S. Sinninghe Damsté

**Affiliations:** 1 NIOZ Royal Netherlands Institute for Sea Research, Department of Marine Microbiology and Biogeochemistry, Utrecht University, P.O. Box 59, 1797AB Den Burg, Texel The Netherlands; 2 Faculty of Geosciences, Department of Earth Sciences, Utrecht University, P.O. Box 80.021, 3508 TA Utrecht, The Netherlands; 3 Theoretical Biology and Bioinformatics, Science for Life, Utrecht University, 3584CH Utrecht, The Netherlands; 4 Centre for Molecular and Biomolecular Informatics, Radboud Institute for Molecular Life Sciences, Radboud University Medical Centre, Utrecht, The Netherlands

**Keywords:** Water microbiology, Cellular microbiology, Molecular evolution, Metagenomics

## Abstract

Archaea synthesize membranes of isoprenoid lipids that are ether-linked to glycerol-1-phosphate (G1P), while Bacteria/Eukarya produce membranes consisting of fatty acids ester-bound to glycerol-3-phosphate (G3P). This dichotomy in membrane lipid composition (i.e., the ‘lipid divide’) is believed to have arisen after the Last Universal Common Ancestor (LUCA). A leading hypothesis is that LUCA possessed a heterochiral ‘mixed archaeal/bacterial membrane’. However, no natural microbial representatives supporting this scenario have been shown to exist today. Here, we demonstrate that bacteria of the Fibrobacteres–Chlorobi–Bacteroidetes (FCB) group superphylum encode a putative archaeal pathway for ether-bound isoprenoid membrane lipids in addition to the bacterial fatty acid membrane pathway. Key genes were expressed in the environment and their recombinant expression in *Escherichia coli* resulted in the formation of a ‘mixed archaeal/bacterial membrane’. Genomic evidence and biochemical assays suggest that the archaeal-like lipids of members of the FCB group could possess either a G1P or G3P stereochemistry. Our results support the existence of ‘mixed membranes’ in natural environments and their stability over a long period in evolutionary history, thereby bridging a once-thought fundamental divide in biology.

## Introduction

Lipid membranes are essential for all cellular life forms to preserve the integrity and individuality of cells, as well as having a direct influence in the maintenance of energy metabolism. Lipid membranes are also key in differentiating the domains of life. Bacteria and eukaryotes have membranes formed by fatty acids linked to glycerol-3-phosphate (G3P) via ester bonds, while archaea have membranes made of isoprenoid alkyl chains linked by ether linkages to glycerol-1-phosphate (G1P), leading to an opposite stereochemistry of the glycerol phosphate backbone [1]. This segregation in lipid membrane composition, or ‘lipid divide’, has been hypothesized to have appeared early in the evolution of microbial life from the Last Universal Common Ancestor (LUCA), but the nature of the lipid membrane of LUCA and its subsequent differentiation in Bacteria and Archaea remain unknown. Some studies have proposed that a non-cellular LUCA lacked a lipid membrane [2–4]. A recent study suggested that the membrane of LUCA was formed by fatty acids and isoprenoids without the glycerol phosphate backbone as a requirement to have a lower membrane permeability that could sustain a proton gradient [5]. The most parsimonious hypothesis may be that LUCA had a heterochiral lipid membrane composed of both G1P and G3P together with fatty acids and isoprenoids [6, 7], which later diversified into archaeal and bacterial membranes resulting in the ‘lipid divide’. It was originally proposed that this differentiation may have been driven by heterochiral membrane instability [8, 9], but heterochiral membranes are in fact stable [10] and a recent study has proven that they are, in some cases, more robust to environmental stresses [11].

Another critical issue in the ‘lipid divide’ is the membrane lipid composition of eukaryotes. Multiple lines of evidence indicate that eukaryogenesis encompassed an endosymbiosis event of a bacterial cell into an archaeal host [12–17]. Thus, the bacterial-like composition of contemporary eukaryotic membranes implies that an early eukaryote had its archaeal-like membrane replaced by a bacterial-like one, possibly through a ‘mixed membrane’ intermediate containing both the archaeal membrane lipids with ether-linked isoprenoids to G1P and the bacterial ones with ester-linked fatty acids to G3P. This would imply that bacterial-like membrane molecules arose twice, in bacteria and in eukaryotes. The competing syntrophic hypothesis of eukaryogenesis [18–20] proposes that the host of the mitochondrial endosymbiont was a bacterium, avoiding the need for a transitional step from an archaeal to eukaryotic membrane. Some eukaryogenesis models also suggest that the membrane transition was facilitated by intensive bacterial lipid transfer from the endomembrane system [21]. Because most models for the origin of eukaryotes require a membrane transitional step similar to the one expected in the ‘mixed membrane’ scenario for LUCA, it is striking that no remnants or natural microbial representatives with a heterochiral ‘mixed membrane’ have yet been described that would support the viability of such a scenario.

The concept of the ‘lipid divide’ has been challenged by the identification of traits thought to be characteristic of archaeal membrane lipids in bacteria and vice versa, mostly restricted to specific taxonomic groups. First, some bacteria (and eukaryotes) produce ether-linked lipids [22–28]. Second, membrane-spanning lipids, another trait thought to be specific for the archaea, also occur in bacteria [27–31]. However, no bacteria are known to produce membrane lipids based on isoprenoidal side chains or possessing the ‘archaeal’ G1P stereochemistry. Third, some studies have reported that some archaea produce phospholipid fatty acids (e.g., [32]) but this may be due to contamination of the growth media [33]. Nevertheless, an almost complete biosynthetic pathway for fatty acid synthesis is encoded by many archaeal genomes [34]. Last, two uncultured archaeal groups, the Euryarchaeota Marine Group II (currently known as *Candidatus* Poseidoniales ord. nov. [35]) and Lokiarchaeota of the Asgard superphylum, contain archaeal lipid biosynthesis genes alongside bacterial-like fatty acid and ester-bond formation genes, but seem to lack the capacity to synthesize the G1P backbone via glycerol-1-P-dehydrogenase (G1PDH), while they do have the genetic ability to produce G3P [36]. This observation is exciting as the Asgard archaea are currently considered as the closest descendants of the archaeal ancestor leading to eukaryotes [37]. However, a recent phylogenomic study of the orthologs of bacterial lipid genes present in Asgard archaea does not provide compelling support for an origin of eukaryotic lipids via an archaeal host cell [38]. Moreover, there is no further evidence to date that the presence of these genes in those two archaeal groups actually leads to the synthesis of ‘mixed membranes’, i.e., membranes containing both ‘bacterial’ fatty acid ester-linked lipids and ‘archaeal’ isoprenoid ether-linked lipids, either heterochiral (containing G1P and G3P) or homochiral. Taken together, these observations expose a ‘lipid divide’ that is not as clear-cut as originally thought. Nonetheless, no natural microbial representatives have ever been reported to synthesize ‘mixed membranes’.

Here, we present the discovery of living bacteria of the phylum *Candidatus* Cloacimonetes of the Fibrobacteres–Chlorobi–Bacteroidetes (FCB) group superphylum, which are highly abundant in the deep anoxic waters of the Black Sea, harboring a putative ‘mixed archaeal/bacterial membrane’. We observed that the metagenome-assembled genomes (MAGs) of this bacterial phylum contain the genes of the canonical bacterial fatty acid biosynthetic pathway but also, unexpectedly, homologs of key enzymes for archaeal membrane lipid biosynthesis. We validated the presence of these protein-coding genes both in silico and experimentally, and observed that they were expressed in the environment. Expression of these genes in *Escherichia* *coli* leads to the formation of a membrane containing ether-linked isoprenoid phospholipids. We hypothesize that the ‘archaeal’ membrane lipids of *Ca*. Cloacimonetes have the G1P stereochemistry, awaiting validation based on isolation of these elusive bacteria and analysis of their membrane lipids. Database searches revealed the presence of the key archaeal membrane lipid biosynthetic genes not only in other *Ca*. Cloacimonetes genomes, but also in other genomes of the FCB group superphylum and related candidate phyla, indicating that the ability to produce ‘mixed membranes’ might be widespread in the tree of life.

## Materials and methods

### Oceanographic sampling

All cruises in the Black Sea western gyre were performed with the R/V Pelagia. Suspended particulate matter (SPM) from 15 depths across the water column (50–2000 m) was collected at sampling station 2 (N42°53.8′, E30°40.7′, 2107 m depth) during the Phoxy cruise 64PE371 (BS2013) on 9–10 June 2013 (Table S1). At the same station, SPM was also collected from 1000, 1500, and 1980-m depth during the NESSC cruise 64PE408 (BS2016) on 31 January–2 February 2016, and from 2000 m depth during the 64PE444 cruise (BS2018) on 17 August 2018. SPM from four depths (500, 1000, 1500, and 2000 m) was collected at sampling station 4 (N42°46.9′, E29°21.1′, 2100 m depth) during the 64PE418 cruise (BS2017) on 27 March–5 April 2017. For the BS2013 and BS2017 cruises, SPM was collected with McLane WTS-LV in situ pumps (McLane Laboratories Inc., Falmouth) on pre-combusted glass fiber filters with 142-mm diameter and 0.7 and 0.3-µm pore size, respectively. For the BS2016 and BS28018 cruises, SPM was collected on 0.22 µm Sterivex cartridge filters (Millipore). In all cases, all samples were stored at −80 °C until nucleic acid or lipid extraction (only for the glass fiber filters) was performed. Water samples were collected during the BS2018 cruise to attempt enrichment cultures and for visualization of the cell morphology as specified in the Supplementary Information.

### Lipid analysis environmental samples

Total lipids were extracted from freeze-dried glass fiber filters as described in [39]. The Bligh and Dyer lipid extracts are expected to contain archaeal intact polar lipids (IPLs), which are composed of the core lipid (CL) attached to one or two polar head groups [40]. The Bligh and Dyer extracts were both analyzed directly for the presence of total archaeal CL, and also after acid hydrolysis to remove the polar head groups and quantify both the archaeal CLs and the IPL-derived CLs. Subtracting CLs from CL + IPL-derived CLs allows for determination of the IPL-derived CLs linked to living archaeal biomass. Acid hydrolysis was performed in nitrogen-dried Bligh and Dyer extracts [41]. Extracts were analyzed by UHPLC–atmospheric pressure chemical ionization MS for archaeol and GDGTs, according to Hopmans et al. [42] with some modifications. Briefly, analysis was performed on an Agilent 1260 UHPLC coupled to a 6130 quadrupole MSD in selected ion monitoring mode. Separation was achieved on two UHPLC silica columns (BEH HILIC columns, 2.1 × 150 mm, 1.7 µm; Waters) in series, fitted with a 2.1 × 5 mm pre-column of the same material (Waters) and maintained at 30 °C. Archaeol and GDGTs were eluted isocratically for 10 min with 10% B, followed by a linear gradient to 18% B in 20 min, then a linear gradient to 100% B in 20 min, where A is hexane and B is hexane: isopropanol (9:1). Flow rate was 0.2 ml min^−1^. Total run time is 61 min with a 20 min re-equilibration. Source settings were identical to Schouten et al. [43]. Typical injection volume was 10 µl of a 1 mg ml^−1^ solution. The *m/z* values of the protonated molecules of archaeol and isoprenoid GDGTs were monitored. Archaeol and GDGTs were quantified by adding a C_46_ GTGT internal standard by using an archaeol:GDGT-0 standard (1:1) to correct for response differences between archaeol and GDGTs [44].

### Nucleic acid extraction and 16S rRNA gene amplicon sequencing

DNA and RNA were extracted from sections of the glass fiber filters (1/8 filter from 50 to 130-m depth and 1/4 from 170 to 2000-m depth) or from the Sterivex filter cartridge with the RNA PowerSoil^®^ Total Isolation Kit plus the DNA elution accessory (Mo Bio Laboratories, Carlsbad, CA). RNA extracts were treated with DNAse and reverse-transcribed to cDNA using random nonamers as described previously [45]. The 16S rRNA gene amplicon sequencing and analysis was performed as described previously [46] (see Table S2 and Supplementary Information for details). Taxonomy of the reads was assigned based on blast [47] and the SILVA database version 123 [48].

### Metagenome sequencing and assembly

Unamplified DNA extracts from the 15 SPM samples of BS2013 were used to prepare TruSeq nano libraries which were further sequenced with Illumina Miseq (5 samples multiplexed per lane) at Utrecht Sequencing Facility, generating 45 million 2 × 251 bp paired-end reads. Quality control was performed with FastQC v0.11.3 (https://www.bioinformatics.babraham.ac.uk/projects/fastqc/) and reads with uncalled bases and remaining TruSeq adapters were removed with Flexbar v2.5 [49], keeping the longer side of the read with the ‘--ae any’ flag. All reads were cross-assembled with SPAdes v3.8.0 in ‘--meta’ mode [50], with read error correction turned on. BWA-MEM v0.7.12-r1039 [51] was used to map the forward and reverse reads from individual samples to the cross-assembled scaffolds.

### Scaffold binning and assessment of MAG quality

Scaffolds were binned into draft genome sequences based on coverage profile across samples and tetra-nucleotide frequency with MetaBAT v0.32.4 [52]. The ‘--superspecific’ preset was used to minimize contamination. To increase sensitivity without losing specificity, MetaBAT was run with ensemble binning, which aims to combine highly similar bins (‘-B’ and ‘--pB’ were set to 20% and 50%, respectively). Quality of the MAGs was assessed based on absence and presence of lineage-specific marker gene sets after genome placement in a reference tree with CheckM v1.0.7 [53] in ‘--lineage_wf’ mode.

### Genome annotation and abundance estimation

MAGs were annotated with Prokka v1.11 [54] in ‘--metagenome’ mode. GenBank output files generated by Prokka were also annotated with the Rapid annotation using subsystem technology pipeline v2.0 [55]. The geranylgeranylglyceryl phosphate (GGGP) synthase of NIOZ-UU2 is only partially predicted by Prokka as it bridges a scaffold boundary (see Supplementary Information). For the homology searches and tree constructions detailed below a longer protein was reconstructed by concatenating the predicted partial protein sequence with the last part of the translated linked scaffold.

**Fig. 1:**
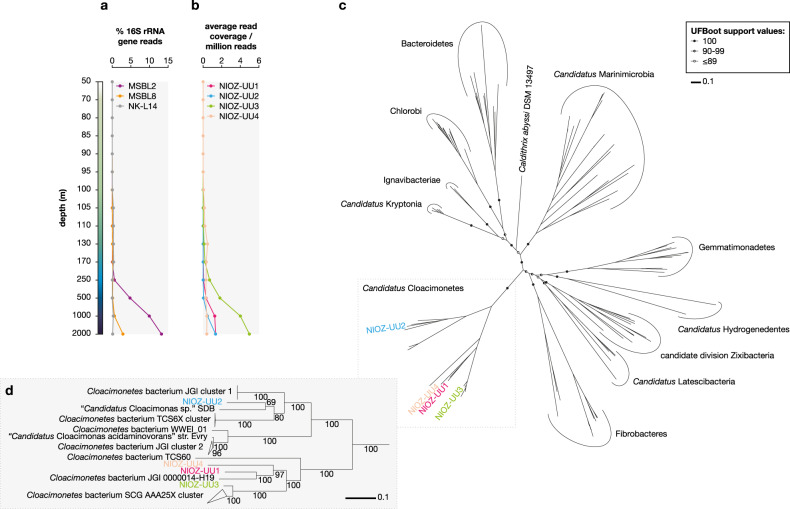
Distribution within the Black Sea water column and phylogeny of *Ca*. Cloacimonetes. **a** Percentage of 16S rRNA gene reads attributed to different *Ca*. Cloacimonetes groups during BS2013. **b** Estimated abundance of the four *Ca*. Cloacimonetes MAGs. **c** Maximum likelihood phylogenetic tree of the FCB group superphylum based on 43 concatenated core genes. Circles along branches indicate ultrafast bootstrap approximation support values, with only values for deepest nodes shown. **d** Zoom in on the *Ca*. Cloacimonetes phylogeny. Numbers along branches indicate ultrafast bootstrap approximation support values. Scale bars in **c**, **d** represent mean number of substitutions per site.

MAG abundance was estimated from the shotgun metagenomics data by generating depth files per sample for all the scaffolds with SAMtools v.1.3.1 [56], using the mpileup utility with flags ‘-aa’ and ‘-A’ (count orphans) set. Average read coverage per nucleobase of a scaffold was calculated by dividing the sum of depth of all positions by the length of the scaffold with N’s removed. Coverage of a MAG was calculated likewise after concatenating the depth files of the scaffolds in that MAG. Average read coverage per nucleobase was normalized across samples by dividing by the total number of reads after quality control in the sample times 1,000,000. Normalized data is only shown in Fig. 1.

### Manual cleaning of the MAGs

The four *Ca*. Cloacimonetes MAGs were cleaned by plotting coverage across samples for all the scaffolds in the MAG, and manually removing those scaffolds that did not clearly have the same coverage profile as the majority (see Supplementary Information for details). NIOZ-UU3 appeared clean based on its coverage profile so none of its scaffolds were removed. Because the removed scaffolds do not contain marker genes, completeness and contamination estimates did not change (see Table S3 for extended CheckM results of the four MAGs). Genome annotations and abundance estimations of MAGs were newly generated as described above after cleaning.

### Placement of MAGs in the FCB group superphylum tree

To further check the phylogenetic affiliation of the four MAGs in comparison with close relatives, Bacteroidetes/Chlorobi group genomes were downloaded from RefSeq [57], and other FCB group genomes from GenBank [58], both on February 8, 2017 (Table S4). For Ignavibacteriae and Chlorobi all the RefSeq representative genomes were downloaded, and for Bacteroidetes only the first ten representative genomes. We only included genomes that were estimated to be less than 10% contaminated by CheckM in ‘--lineage_wf mode’, and that contained at least 4 out of 43 phylogenetically informative marker genes in single copy that CheckM uses for bin placement [53] (Table S4). We realigned the 43 marker genes individually with Clustal Omega v1.2.3 [59]. The genes were concatenated, gaps included if a gene was not present, and identical sequences collapsed (Table S4). A maximum likelihood tree was inferred with IQ-TREE v1.6.3 [60]. Model selection of nuclear models was performed with ModelFinder [61] and the best-fit model (LG + R8) chosen according to Bayesian Information Criterion. Branch support was based on 1000 ultrafast bootstraps [62]. The tree was visualized in iTOL [63].

We identified 16S rRNA genes in the FCB group genomes included in the tree with the CheckM ‘ssu_finder’ utility. 16S rRNA gene sequences were found in 21 *Ca*. Cloacimonetes genomes, including one of the MAGs (NIOZ-UU1) (Table S4). We aligned these sequences with the *Ca*. Cloacimonetes 16S rRNA gene amplicon sequences using MAFFT v7.394 [64], sliced out the amplicon region from the genome sequences, and removed identical sequences (Table S4).

### Homology searches of GGGP synthase and (*S*)-2,3-di-O-geranylgeranylglyceryl phosphate (DGGGP) synthase in the Black Sea water column

Predicted GGGP and DGGGP synthases from the four MAGs were queried with tblastn v2.6.0+ [47, 65] (*e*-value <1*e*^−5^ and ≥70% query coverage) against all scaffolds in the assembly. For the scaffolds with significant hits (61 for GGGP synthase and 43 for DGGGP synthase), we extracted the aligned part of the subject sequence of the best hit based on *e*-value on each scaffold. In addition to the queries we included a set of known GGGP and DGGGP synthases based on biochemical evidence and phylogenetic analyses [66], as well as other non-DGGGP prenyltransferases as ‘outgroups’ (see Supplementary Information). We also queried the GGGP and DGGGP synthases from the four MAGs against the NCBI nonredundant protein database (nr) [67] with blastp [47], and added the four best hits for each gene, all of which were found in *Ca*. Cloacimonetes.

The protein sequences were aligned with MAFFT v7.394 [64], using a maximum number of 1000 iterative refinements and local pair alignment (L-INS-i). The sequence alignments were trimmed with trimAl v1.4.rev22 [68] in ‘-gappyout’ mode, and identical sequences were collapsed. Final alignment lengths were 171 and 200 positions for GGGP synthase and DGGGP synthase, respectively. Maximum likelihood trees were inferred with IQ-TREE. Model selection of nuclear models was performed with ModelFinder and the best-fit model (LG + R7 for GGGP synthase and LG + F + R7 for DGGGP synthase) chosen according to Bayesian Information Criterion. Branch support was based on 1000 ultrafast bootstraps. Trees were visualized in iTOL.

Taxonomy was assigned to hits based on taxonomic classification of the scaffolds on which they were found with Contig Annotation Tool v5.1.2 [69]. Database files were constructed on March 4, 2020. We used Prodigal v2.6.3 [70] for gene prediction and DIAMOND v0.9.21 [71] for protein alignment to nr. The *f* parameter was set to 0.3 to allow for speculative classifications. One scaffold had multiple classifications, and we chose the lowest classification that reached a majority vote based on bit-score support in this case.

### Homology searches of GGGP synthase and DGGGP synthase across the tree of life

Predicted GGGP and DGGGP synthases from the four MAGs were queried with blastp v2.6.0+ [47, 65] against 110,421 annotated genomes available in the PATRIC genome database [72] on November 29, 2017. Blastp was run per genome (*e*-value <1*e*−10 and ≥70% query coverage) with a fixed database size of 20,000,000 to make *e*-values comparable across genomes. For GGGP synthase, we collected all hits and included the entire protein in the analysis. For DGGGP synthase, we only included hits that were annotated as ‘Digeranylgeranylglyceryl phosphate synthase (EC 2.5.1.42)’, and (‘similar to’) ‘(S)−2,3-di-O-geranylgeranylglyceryl phosphate synthase’, and hypothetical proteins with ≥90% query coverage, to exclude other prenyltransferases from the superfamily. Again, entire proteins were included in the analysis. We added the queries, and the set of extra sequences (see above): known GGGP and DGGGP synthases, outgroups, and four best hits for each gene from nr. Identical sequences were collapsed.

Alignment and tree inference were performed with MAFFT, trimAl, and IQ-TREE as described above. Final alignment lengths were 246 and 266 positions for GGGP synthase and DGGGP synthase, respectively. The best-fit models were LG + R10 for GGGP synthase and LG + F + R10 for DGGGP synthase. For both trees, the consensus tree had a higher likelihood than the maximum likelihood tree found. Major clade separations were comparable between the maximum likelihood and consensus tree for both genes. Consensus trees were visualized in iTOL.

### Co-localization of GGGP and DGGGP synthase in FCB group superphylum genomes

Predicted GGGP and DGGGP synthases from the four *Ca*. Cloacimonetes MAGs were queried with tblastn (*e*-value <1*e*^−5^ and ≥70% query coverage) against all downloaded GenBank/RefSeq FCB group superphylum genomes (see above) to identify homologs. If hits were located on the same scaffold, the minimum base pair distance between GGGP and DGGGP synthase homologs was considered as a measure of co-localization.

### Amplification, sequencing, gene expression, and quantification of specific genes in NIOZ-UU3

To experimentally assess the assembly accuracy of NIOZ-UU3, primers were designed to amplify and sequence the region of the scaffold that contains the genes predicted to code for GGGP synthase, DGGGP synthase, polyprenyl synthase, and the bacterial marker gene predicted by CheckM (helicase PriA, see Supplementary Information) from the BS2013 2000-m depth sample. PCR reaction mixture was the following (final concentration): Q-solution (PCR additive) 1×; PCR buffer 1×; bovine serum albumin (200 μg ml^−1^); dNTPs (40 μM); primers (0.4 pmol); MgCl_2_ (1.5 mM); 2.5 U Taq polymerase (Qiagen, Valencia, CA, USA). PCR conditions were the following: 95 °C, 5 min; 35 × (95 °C, 15 s; 62 °C (melting temperature verified by gradient PCR), 30 s; 72 °C, 1 min per kilobase); final extension 72 °C, 5 min. PCR product was gel purified (QIAquick gel purification kit, Qiagen), cloned in the TOPO-TA cloning^®^ kit from Invitrogen (Carlsbad, CA, USA), and sequenced for verification. In addition, to determine the presence and expression of the GGGP synthase, DGGGP synthase, and polyprenyl synthase coding genes, PCRs targeting a fragment of the genes (see primers in Supplementary Information) were tested with DNA and cDNA from the SPM samples recovered at 1000 and 2000-m depth from the Black Sea 2013 campaign as a template with the same PCR master mix as described above but with half amount of dNTPs and Taq Polymerase (melting temperature 62 °C verified by gradient PCR). Moreover, quantitative PCRs (qPCR) with the same specific primers targeting the GGGP and the DGGGP synthase coding genes were performed on DNA and cDNA extracts of the Black Sea 2013 campaign from 50 to 2000-m depth (15 samples). qPCR master mix was the same as used for amplifications above with the addition of Evagreen fluorescent nucleic acid dye (0.625 nM final concentration). PCR analyses were performed on a Bio-Rad CFX96 Real-Time System/C1000 Thermal cycler equipped with CFX Manager Software. Standard curves were generated by dilution of the verified amplicon of the GGGP and DGGGP synthase coding gene fragment obtained with DNA extracts of the SPM at 2000-m depth as described above.

### Construction of GGGPS and DGGGPS expression plasmids

Plasmids for in vivo lipid production were constructed by PCR amplifying the GGGP synthase ORF from pLVA01 and the plasmid pRSFDuet-1 (Novagen) using the primers GGGPS-RSFDuet-F/R and RSFDuet-GGGPS-F/R, respectively, and further recombined [73] (Supplementary Information). A non-synonymous mutation (Met to Val, based on NIOZ-UU3) was corrected by site-directed mutagenesis using the primers GGGPS-SDM-F/R, resulting in pABW1. The DGGGP synthase ORF was amplified using the primers DGGGPS-F/R and cloned into pRSFDuet-1 using NcoI and BamHI to construct pABW2. pABW3 (encoding both GGGPS and DGGGPS) was constructed by re-amplifying DGGPS from pABW2 and cloning the amplicon as a NcoI-BamHI fragment into pABW1. A N-terminally 6His-tagged GGGP synthase overproduction plasmid (pABW4) was constructed by recombining [73] the GGGP synthase ORF from pABW1 and pET-28a(+) (Novagene) amplified using the primers GGGPS-ET28-F/R and ET28-GGGPS-F/R, respectively. All inserts were verified to encode correct proteins by sequencing.

### Recombinant production, purification, and in vitro enzyme assay of GGGP synthase

Purification and enzymatic assay of the NIOZ-UU3 GGGP synthase was based on the method of Jain et al. [74]. *E. coli* BL21(DE3) harboring pABW4 was cultured in 250 ml Lysogeny broth (LB) medium (37 °C, 200 rpm) and induced with 0.5 mM isopropyl β-D-1-thiogalactopyranoside (IPTG) at 0.6 OD_600nm_ for 4 h. Cells were centrifuged (3500 rcf) and frozen. Subsequent steps were performed at room temperature, with samples and buffers kept on ice. Thawed cells were resuspended in ~5 ml lysis buffer (50 mM Tris-HCl pH 7.5, 150 mM NaCl, 20 mM imidazole, 1 mg ml^−1^ lysozyme), and a protease inhibitor (cOmplete™, EDTA-free; Roche, Basel) and sonicated to facilitate cell lysis. The lysate was cleared by centrifugation (20,000 rcf, 10 min), glycerol was added (10% final), and sample loaded onto a gravity column containing 1.5 ml of nickel-nitrilotriacetic acid agarose beads (Qiagen, Venlo, NL) pre-equilibrated with protein buffer (50 mM Tris-HCl pH 7.5, 150 mM NaCl, 20 mM imidazole, 10% glycerol). Beads were washed with ~30 column volumes of protein buffer, and initially eluted by a titration of imidazole with GGGP synthase protein eluting at 200 mM. Subsequent elutions were performed with 250 mM imidazole. The purity of the GGGP synthase protein was verified using 12% TGX^TM^ precast gels (Bio-Rad), stained with Bio-Safe™ Coomassie stain (Bio-Rad). The concentration of the purified protein was calculated based on the absorbance at 280 nm using a predicted extinction coefficient of 9002 M^−1^ cm^−1^ (ExPASy ProtParam, averaged Cys reduced, and cysteine form).

Enzymatic activity of the purified *Ca*. Cloacimonetes GGGP synthase was determined in an end-point assay using 0.1 µM GGGP synthase, 10 mM G1P or G3P and 100 µM geranylgeranylpyrophosphate in a reaction buffer consisting of 50 mM Tris-HCl (pH 7.5), 10 mM MgCl, and carryover amounts of imidazole (1 mM) and glycerol (0.5%). Reactions were incubated for 2 h at 37 °C in glass vials, extracted twice with 300 µL n-butanol (water saturated), pooled and stored at −20 °C. Analysis of the GGGP synthase enzyme assays were performed using ultra-high-pressure liquid chromatography—high resolution mass spectrometry (UHPLC-HRMS) based on Sturt et al. [40]. with some modifications as detailed below. Pooled butanol extracts were evaporated under a stream of nitrogen, redissolved in 50 µL methanol:dichloromethane (1:1) and filtered (0.45 µm, regenerated cellulose). Analysis was performed using an Agilent 1290 Infinity I UHPLC, equipped with thermostatted auto-injector and column oven, coupled to a Q Exactive Orbitrap MS with Ion Max source with heated electrospray ionization (HESI) probe (Thermo Fisher Scientific, Waltham, MA). Injection volume was 1 µl (out of 50 µl). Separation was achieved on a YMC-Triart Diol-HILIC column (250 × 2.0 mm, 1.9 µm particles, pore size 12 nm; YMC Co., Ltd, Kyoto, Japan) maintained at 30 °C. The following elution program was used with a flow rate of 0.2 ml min^−1^: 100% A for 5 min, followed by a linear gradient to 66% A: 34% B in 20 min, maintained for 15 min, followed by a linear gradient to 40% A:60% B in 15 min, followed by a linear gradient to 30% A:70% B in 10 min, where A = hexane/2-propanol/formic acid/14.8 M NH_3aq_ (79:20:0.12:0.04 [volume in volume in volume in volume, v/v/v/v]) and B = 2-propanol/water/formic acid/14.8 M NH_3aq_ (88:10:0.12:0.04 [v/v/v/v]). HESI settings were as follows: sheath gas (N_2_) pressure 35 (arbitrary units), auxiliary gas (N_2_) pressure 10 (arbitrary units), auxiliary gas (N2) T 50 °C, sweep gas (N_2_) pressure 10 (arbitrary units), spray voltage 4.0 kV (positive ion ESI), capillary temperature 275 °C, S-Lens 70 V. Lipids were analyzed with a mass range of *m/z* 350–2000 (resolving power 70,000) followed by data dependent MS^2^ (resolving power 17,500), in which the ten most abundant masses in the mass spectrum (with the exclusion of isotope peaks) were fragmented successively (stepped normalized collision energy 15, 22.5, 30; isolation window 1.0 *m/z*). An inclusion list was used with a mass tolerance of 3 ppm, targeting the ammoniated molecule [C_46_H_79_O_8_P + NH_4_]^+^ of GGGP at *m/z* 462.2979. The Q Exactive was calibrated within a mass accuracy range of 1 ppm using the Thermo Scientific Pierce LTQ Velos ESI Positive Ion Calibration Solution (containing a mixture of caffeine, MRFA, Ultramark 1621, and *N*-butylamine in an acetonitrilemethanol-acetic solution). Identification of GGGP was aided by the analysis of 1-O-octadecyl-2-hydroxy-*sn*-glycero-3-phosphate (C_18_-LPA; Avanti Polar Lipids, Inc. Alabama, USA), a structural analog of GGGP, which has a C_18_ carbon chain attached to the glycerol backbone instead of the geranylgeranyl carbon chain present in GGGP. C_18_-LPA showed similar chromatographic and mass spectral behavior to GGGP.

### Co-expression of GGGP and DGGGP synthases in *E. coli*

*E. coli* C43(DE3) [75] harboring geranylgeranyl diphosphate (GGPP) synthase (crtE) and G1PDH (araM) on plasmid pMS148 [11] was used for expression of the GGGP and DGGGP synthases (encoded on plasmids pABW1, -2 and -3). Cells growing exponentially in LB medium were diluted into magnesium-supplemented terrific broth medium [76] (Mg-TB; 1.2% tryptone, 2.4% yeast extract, 0.4% glycerol, 2 mM MgSO_4_, 0.23% KH_2_PO_4_, and 1.25% K_2_HPO_4_) to 0.01 OD_600nm_ induced with 0.4 mM IPTG and incubated at 37 °C (200 rpm) for 16 h. Cells (10 ml culture normalized to 1.9 OD_600nm_) were harvested by centrifugation (4000 rcf, 10 min) and washed twice with 0.85% NaCl, lyophilized, and then stored at −80 °C. Analysis for production of archaeal-like lipids in *E. coli* was performed by extracting IPLs by a modified Bligh-Dyer extraction [77] that was analyzed according to [29] with some modifications: lyophilized cells were extracted 3× with BDE solvent mixture (2:1:0.8) methanol:dichloromethane (DCM):potassium phosphate buffer (50 mM, pH 7) aided by sonication and centrifugation. The extracts were pooled and solvent ratios adjusted to 1:1:0.9, vigorously mixed, centrifuged, and the lower DCM phase transferred. The upper fraction was re-extracted twice with DCM, and the pooled extract was evaporated under a stream of nitrogen and stored dry at −20 °C until analysis. For analysis, samples were dissolved in 200 µl hexane:isopropanol:H_2_O (718:271:10) and filtered (0.45 µm), regenerated cellulose. Analysis was performed on an Agilent 1200 series LC (Agilent, San Jose, CA), equipped with thermostatted auto-injector and column oven, coupled to a Thermo LTQ XL linear ion trap with Ion Max source with electrospray ionization (ESI) probe (Thermo Scientific, Waltham, MA), was used. Separation was achieved on a YMC-Pack-Diol-120-NP column (250 × 2.1 µm, 5 µm particles; YMC Co., Ltd, Japan) maintained at 30 °C. Elution program and ESI settings are described in [29]. The lipid extract was analyzed by an MS routine where a positive ion scan (m/z 400–2000) was followed by a data dependent MS^2^ experiment where the base peak of the mass spectrum was fragmented (normalized collision energy (NCE) 25, isolation width 5.0, activation Q 0.175). This was followed by a data dependent MS^3^ experiment where the base peak of the MS^2^ spectrum was fragmented under identical fragmentation conditions. This process was repeated on the 2nd–4th most abundant ions of the initial mass spectrum.

## Results and discussion

### An abundant bacterium in the anoxic water column of the Black Sea

The microbial diversity in the water column of the Black Sea, a basin whose euxinic waters may resemble the ancient oceans [78], was determined by 16S rRNA gene amplicon sequencing (Tables S1 and S2). Remarkably, a group of bacteria attributed to *Ca*. Cloacimonetes was very abundant (i.e., representing 5–20% of the total bacterial plus archaeal 16S rRNA gene reads) in the euxinic waters between 500 and 2000-m depth (Fig. 1a and Table S2). Catalyzed reporter deposition Fluorescence In Situ Hybridization (CARD-FISH) using a specific probe targeting *Ca*. Cloacimonetes confirmed their presence in the deep Black Sea waters (i.e., 2000-m depth) and identified the cell morphology of the *Ca*. Cloacimonetes cells as oval to small rods (Fig. S1).

A genome-centric metagenomics approach was subsequently undertaken to shed light on the physiology of *Ca*. Cloacimonetes; four draft genomes of this group with substantial to near completeness and low contamination were assembled (see Supplementary Information; Table S3). The high abundance of these MAGs in the deep waters matched the *Ca*. Cloacimonetes 16S rRNA gene abundance profile (Fig. 1b). Phylogenomic analysis based on 43 concatenated core genes confirmed their taxonomic position (Fig. 1c, d and Fig. S2). Based on their genomic content, the predicted metabolism of the *Ca*. Cloacimonetes found in the Black Sea is compatible with polysaccharide hydrolysis and fermentation of sugars and amino acids under an anaerobic or microaerophilic lifestyle (see Supplementary Information). Based on this genomic information, six different growth media supplemented with cellulose, cellobiose, microbial cell lysate, and amino acid mix were tested to obtain an enrichment but cultivation of *Ca*. Cloacimonetes was not achieved, possibly because culture conditions did not mimic the high hydrostatic pressure present in the deep Black Sea water column (see Supplementary Information for details).

### An archaeal membrane lipid biosynthetic pathway encoded by the *Ca*. Cloacimonetes genomes

The analysis of the MAGs revealed unexpected features in the lipid biosynthetic pathways of *Ca*. Cloacimonetes. Genes encoding the canonical bacterial fatty acid biosynthetic pathway were detected, including the gene (gps) coding for glycerol-3-phosphate dehydrogenase (catalyzing the formation of G3P), the acyltransferases responsible for the esterification of fatty acids and G3P, as well as genes coding for enzymes involved in downstream reaction [79] (see Supplementary Information). Hence, *Ca*. Cloacimonetes harbors the genes enabling the formation of a normal bacterial membrane (i.e., based on G3P-esterified fatty acids). Strikingly, however, putative key genes of the archaeal lipid biosynthetic pathway (Fig. S3) were also detected in all four *Ca*. Cloacimonetes MAGs. The gene encoding GGGP synthase is co-localized (i.e., encoded in close proximity) with the gene encoding DGGGP synthase (Supplementary Information). Homology searches show that the two genes are found in other Bacteria and Archaea in the Black Sea water column as well (Figs. S4 and S5). These two presumed archaeal enzymes together mediate the formation of the two ether bonds between isoprenoid alcohols and G1P resulting in the production of unsaturated archaeol [1]. Distant homologs of both GGGP and DGGGP synthase genes have previously been reported in bacterial genomes, however, their presence has never been associated with the production of ‘archaeal’ lipids. Whereas GGGP synthase activity has been confirmed in only a few bacteria [80], DGGGP synthase belongs to a large superfamily of UbiA prenyltransferases with several different potential functions [34] and bacterial homologs are assumed to have a different function. Moreover, there has never been an indication for co-expression of the two genes in bacteria. We tested and rejected the possibility that the co-localized GGGP and DGGGP synthase homologs in the *Ca*. Cloacimonetes MAGs could have been introduced by a methodological error, both by in silico analyses and by experimentally amplifying and resequencing one of the scaffolds containing these genes from the original Black Sea water (Supplementary Information, Figs. S6 and S7). The presence of *Ca*. Cloacimonetes GGGP and DGGGP synthase gene transcripts in the Black Sea water column confirmed that they were also expressed in the environment (Supplementary Information, Figs. S8–S10). This resulted in the hypothesis that this bacterium uses the GGGP and DGGGP synthases actively and thus is capable of synthesizing archaeal-like membrane lipids.

**Fig. 2:**
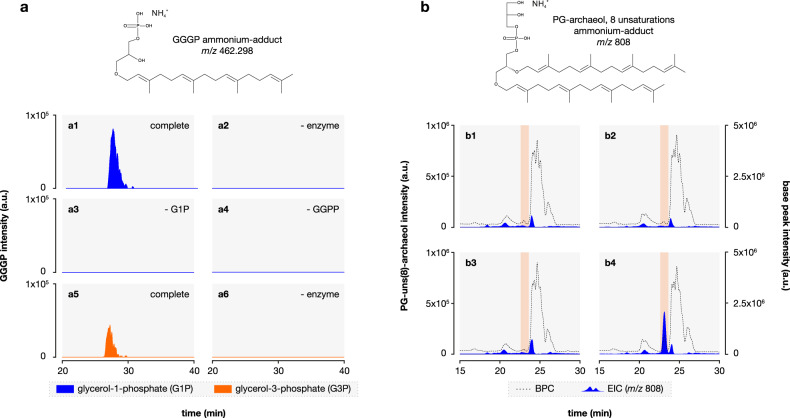
Biochemical verification of *Ca*. Cloacimonetes GGGP and DGGGP synthase activities. **a** Enzyme assay of purified GGGP synthase with G1P (a1–a4) or G3P (a5 and a6). Extracted ion chromatogram, within 3 ppm mass accuracy, of [GGGP + NH_4_]^+^ (*m/z* 462.298) of complete enzymatic assay (a1 and a5) or control assays lacking enzyme (a2 and a6), glycerol phosphate (a3) or geranylgeranyl-diphosphate, GGPP (a4). **b** Production of the lipid PG-archaeol with eight double bonds or unsaturations (PG-unsat(8)-archaeol) in *E. coli*. Extracted ion chromatogram (±0.5, mass units, mu) of [PG-unsat(8)-archaeol+H]^+^ (*m/z* 808); (blue filled area, left axis) or base peak chromatogram (dotted line, right axis) of optimized *E. coli* containing empty-vector (b1) or vector encoding GGGP synthase (b2), DGGGP synthase (b3), or both GGGP and DGGGP synthases (b4). Orange boxes spans the retention time of PG-unsat(8)-archaeol.

Next, we set out to verify that the detected genes coding for putative GGGP and DGGGP synthase have the predicted activity leading to archaeal-like membrane lipids. Previous studies have determined that GGGP synthase homologs of the phylum Bacteroidetes have in vitro GGGP synthase activity with G1P, like the archaeal GGGP synthase [80], rather than the heptaprenyl synthase activity of the bacterial PcrB orthologs detected in *Bacillus subtilis* [81]. To biochemically verify the predicted enzymatic activity in *Ca*. Cloacimonetes, we recombinantly produced its GGGP synthase protein in *E. coli*. The purified protein was found to catalyze formation of GGGP from GGPP in an enzymatic assay (Fig. 2a; Supplementary Information, Figs. S11–S13), as expected in the archaeal membrane lipid biosynthetic pathway [1]. Then, we tested if the *Ca*. Cloacimonetes GGGP and DGGGP synthases could support the formation of a ‘mixed membrane’ in a bacterial cell, by co-expressing them in an *E. coli* optimized for production of GGPP and G1P, the likely substrates of the two enzymes. Cells producing both GGGP and DGGGP synthase contained significant amounts of phosphatidylglycerol (PG) archaeol (i.e., archaeol, also known as diphytanyl glycerol diether) with PG as a polar head group with eight double bonds (Fig. 2b; Supplementary Information, Fig. S14), the expected intermediate in the biosynthesis of archaeal membrane lipids in the absence of a specific geranylgeranyl reductase in *E. coli* [82, 83]. Hence, these experiments provide strong evidence for potential synthesis of ether-linked isoprenoid phospholipids by this group of bacteria.

### Additional lipid biosynthetic genes in the *Ca*. Cloacimonetes MAGs

In addition to genes for GGGP and DGGGP synthase, other genes required for the synthesis of isoprenoidal archaeal lipids (Fig. S3) were also detected in the *Ca*. Cloacimonetes MAGs. They contain the genes for a complete bacterial isoprenoid MEP/DOXP pathway, genes coding for acetyl-CoA C-acetyltransferase and hydroxymethylglutaryl-CoA synthase of the Mevalonate pathway, and two polyprenyl synthases (see Supplementary Information, Fig. S15a), which support the existence of the biosynthetic pathway leading to GGPP, one of the substrates of GGGP synthase. Finally, the MAGs also encode geranylgeranyl reductases that are closely related to homologs found in Euryarchaeota (see Supplementary Information, Fig. S15b). Thus, in addition to ether bond formation (mediated by the putative GGGP and DGGGP synthases), these bacteria have the capacity to synthesize isoprenoid chains (via isoprenoid biosynthetic pathways and the presence of polyprenyl synthases) and saturate them (via the putative geranylgeranyl reductases), characteristics that are fully in line with archaeal lipid membrane biosynthesis [1].

Only two genes of the known lipid biosynthetic pathway in archaea were not detected in the *Ca*. Cloacimonetes MAGs. One is the CDP-archaeol synthase (CarS) forming the activated CDP-archaeol before the addition of the polar head groups in combination with other specific archaeal enzymes [8]. However, it has been recently demonstrated that the bacterial CDP diacylglycerol synthase can replace the function of the archaeal CarS to generate CDP-archaeol [84]. Subsequently, substrate promiscuity allows the bacterial phosphatidylglycerophosphate synthetase together with the phosphatidyl­glycerophosphatase to recognize CDP-archaeol and synthesize archaetidylglycerol [84] as in the archaeal lipid biosynthetic pathway. All these bacterial enzymes are encoded by the four MAGs, which together with the biosynthetic genes mentioned above further support the formation of archaeal-like membrane lipids.

The second gene that is not detected in the four MAGs is the gene (egsA) coding for the enzyme enabling G1P biosynthesis (i.e., G1PDH). Its bacterial homolog (araM), occurring in a few bacteria [85], is also absent. This seems enigmatic at first sight, as it would suggest that the presumed archaeal membrane lipids synthesized by *Ca*. Cloacimonetes do not have G1P as a glycerol phosphate backbone. Rather, they could have the G3P stereochemistry as promiscuity of GGGP synthase for G3P has been observed by others [11, 86, 87], as well as in our enzyme assay (Fig. 2a). However, it was recently demonstrated that an alternative pathway for the formation of G1P must exist within bacteria [11]. Notably, a genetically engineered bacterial strain of *E. coli*, whose genome contained archaeal lipid biosynthesis genes, formed archaeal membrane lipids with the G1P stereochemistry even when the araM coding gene was not included in the genetic construct. The genome did not contain egsA either. This shows that araM is not required for G1P synthesis in *E. coli*. This study also highlighted that in the presence of G3P as substrate, there is still a very high stereoselectivity towards G1P for the formation of archaeal membrane lipids. While we do not know how *E. coli* produces G1P, it is striking that the only known archaeal lipid biosynthesis gene that is missing in the *Ca*. Cloacimonetes MAGs is nonessential for the formation of archaeal-like membrane lipids.

In summary, the four *Ca*. Cloacimonetes MAGs encode a complete set of genes for the synthesis of ether-linked isoprenoid phospholipids. When archaeal GGGP and DGGGP synthase coding genes were previously expressed in *E. coli*, it formed a heterochiral membrane with G3P-bacterial lipids and G1P-archaeal lipids [11]. Given the strong stereoselectivity of the archaeal membrane lipid pathway for G1P and the observation that within bacteria an unknown G1P synthesis pathway exists, we hypothesize that *Ca*. Cloacimonetes also forms ether-linked isoprenoid phospholipids with the G1P stereochemistry, which should be confirmed with the isolation and lipid analysis of members of this phylum.

The presence of the above-mentioned genes encoded by the *Ca*. Cloacimonetes MAGs are compatible with the formation of archaeol (diether) lipid membranes. While many archaeal groups have been seen to synthesize also or exclusively tetraether membrane lipids (i.e., glycerol dibiphytanyl glycerol tetraethers, GDGTs; [88]), the genes required for their synthesis (i.e., potential GDGT synthase) remain unknown, thus we are unable to determine if the *Ca*. Cloacimonetes MAGs could synthesize tetraether membrane lipids.

### Estimating the *Ca*. Cloacimonetes contribution to the ‘archaeal’ membrane lipid pool in the Black Sea

Next, we assessed whether the observed *Ca*. Cloacimonetes abundances could significantly contribute to the ‘archaeal’ membrane lipids of living cells. In order to do so, archaeal IPLs (i.e., archaeol and GDGTs) were quantified in the Black Sea deep water column. IPLs are relatively easily hydrolyzed once the cell dies and therefore considered as biomarkers of living biomass [89].

**Fig. 3:**
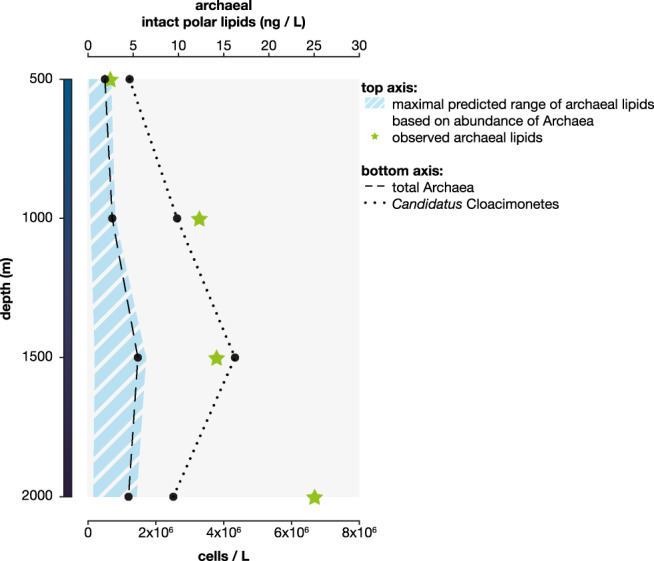
Detected archaeal intact polar lipids (IPLs) in nanograms per liter (green stars) in the Black Sea water column, and predicted archaeal IPLs (shaded area) considering different estimates of membrane lipid production and cell size for the archaeal groups found. Total archaeal cells and *Ca*. Cloacimonetes cells per liter based on qPCR and amplicon sequencing data are also indicated. All calculations are available in Supplementary File 1.

We estimated the absolute abundance of archaeal IPLs, which showed an increase from 2.5 to 25 ng L^−1^ from 500 to 2000-m depth (Fig. 3). The archaeal population in the euxinic waters of the Black Sea mostly consists of Bathyarchaeota, DPANN archaea, and Euryarchaeota Thermoplasmatales, and ANME-1 (Table S2B, see also [39]). Based on their estimated cell numbers in the water column and their expected lipid content per cell we predict a maximum archaeal membrane lipid concentration of <6.5 ng L^−1^ (Fig. 3; Supplementary Information). At 1000–2000-m depth, this represents a striking offset between observed and expected archaeal IPL concentrations ranging at least from two- to fivefold. The mismatch between observed and expected is likely larger considering that these estimates represent an ideal case scenario (see Supplementary Information). The mismatch could be due to preservation of suspended IPLs in the anoxic waters of the Black Sea. Extracellular archaeal IPLs, especially those with more stable glycosidic bonds, could potentially be preserved as fossils as previously observed in deep anoxic sediments (e.g., [90]). However, this possibility seems unlikely, as the IPLs are detected in the water column, which is a more dynamic system than sediments. Moreover, most of the archaeal IPLs detected in the Black Sea do not have glycosidic-based polar head groups but the more labile PG, phosphatidylserine, or phosphatidylethanolamine head groups [39]. Therefore, the mismatch is more likely explained by production of archaeal-like ether-linked isoprenoid membrane lipids by the highly abundant *Ca*. Cloacimonetes bacteria. It is currently not possible to determine the stereochemistry of the archaeal IPLs detected in environmental samples as the analysis would require much higher concentrations of purified archaeal lipids. Hence, although experimental confirmation of archaeal IPL synthesis by *Ca*. Cloacimonetes bacteria and the stereochemistry of these lipids await their isolation, these environmental observations provide enticing circumstantial evidence for the bacterial production of archaeal-like membrane lipids.

### Archaeal lipid biosynthetic genes in *Ca*. Cloacimonetes and other bacteria

**Fig. 4:**
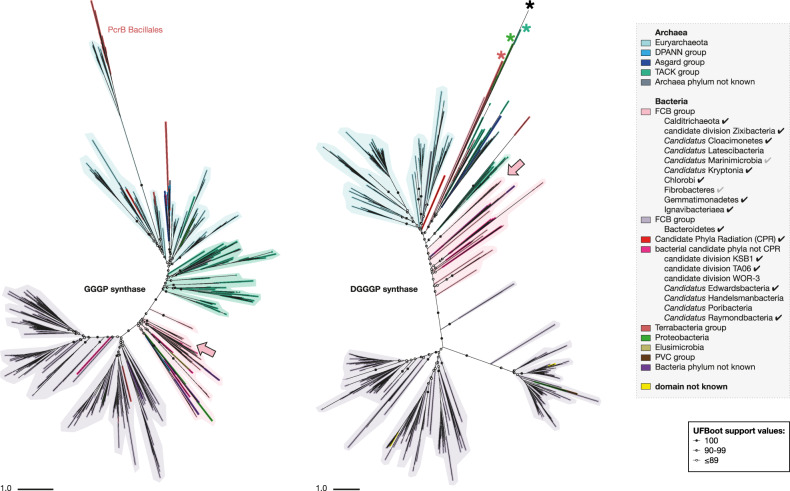
Phylogenetic trees of the GGGP and DGGGP synthase homologs detected across the tree of life. Search for homologs was performed with *Ca*. Cloacimonetes MAG sequences (arrows), in genomes from both cultures and environmental samples. Shadings illustrate the dominant group in the clade. Circles along branches indicate ultrafast bootstrap approximation support values, with only values for deepest nodes shown. See Fig. S6 and Fig. S7 for annotated trees with all branch support values. Scale bars represent mean number of substitutions per site. Asterisks in the DGGGP synthase tree indicate known UbiA prenyltransferases that do not have DGGGP synthase activity. A checkmark in the legend marks bacterial groups with genomes that code for both GGGP and DGGGP synthase, a gray checkmark marks groups in which both genes are found but not in the same genome. TACK includes the Thaumarchaeota and the Crenarchaeota. Archaeal or bacterial ‘phylum not known’: phylum is not known but the genome is annotated on a lower level, or sequence represents multiple groups. ‘Domain not known’: genomes for which no lineage was found on the PATRIC servers.

Homology searches coupled to phylogenetic analyses indicated that the presence of archaeal lipid biosynthetic genes in bacterial genomes is not limited to *Ca*. Cloacimonetes from the Black Sea. Close GGGP and DGGGP synthase homologs are found together in other genomes of *Ca*. Cloacimonetes, as well as in other FCB group superphylum bacteria, related bacterial candidate phyla, and in a genome of *Ca*. Parcubacteria of the Candidate Phyla Radiation [91] (Fig. 4; Figs. S16–S18, Supplementary Information). The phylogenetic topologies of both the GGGP and DGGGP synthases in bacteria are similar with respect to both branching of bacterial groups and in sharing a close affiliation with GGGP and DGGGP synthases from the archaeal TACK group, in particular Crenarchaeota [34], suggesting that the two genes share a similar evolutionary history (Fig. 4; Figs. S16 and S17, Supplementary Information). Co-localization of GGGP synthase and DGGGP synthase as observed in the four *Ca*. Cloacimonetes MAGs has previously only been seen in some Euryarchaeota, and within bacteria seems to be restricted to *Ca*. Cloacimonetes (Supplementary Information). Considering the basal placement of these genomes in the bacterial clade of both trees (Fig. 4, Figs. S16 and S17), we hypothesize that genomic co-localization of the genes is the ancestral state.

The extended presence and close phylogenetic associations of the GGGP and DGGGP synthase homologs in the FCB group superphylum and related candidate phyla strongly supports an origin in Bacteria before the radiation of the FCB group superphylum. This suggests that the capacity to synthesize ‘mixed membranes’ has been a trait of these bacteria for a long period in evolutionary history. Two evolutionary scenarios may explain our current observations. First, the contemporary presence of these enzymes in the FCB group superphylum bacteria could reflect an evolutionary remnant of the ‘mixed membrane’ stage after LUCA but before the diversification of Bacteria. Notably, a recent study that also evaluated the phylogeny of GGGP and DGGGP synthases using outgroup-free routing could not exclude a LUCA origin for both genes [38]. Second, if the ancestor of the FCB group superphylum contained a homochiral bacterial membrane like the other contemporary bacterial groups, the phylogenetic similarities of the co-localized genes could also reflect an ancient horizontal gene transfer (HGT) from an ancestral archaeal lineage into the FCB group ancestor (see Supplementary Information).

### Implications for the ‘lipid divide’

In contrast to the dogma of the ‘lipid divide’, bacteria of the FCB group superphylum harbor a complete archaeal-like membrane lipid biosynthetic pathway. Our data indicate that members of *Ca*. Cloacimonetes have the potential to synthesize ether-linked isoprenoid phospholipids, possibly with the G1P stereochemistry, in which case they would possess a true heterochiral ‘mixed membrane’. This is the first evidence of naturally occurring organisms with this ability. The presence of bacterial and archaeal lipid biosynthesis genes in *Ca*. Cloacimonetes strikingly resembles the Marine Group II Euryarchaeota (currently known as *Candidatus* Poseidoniales ord. nov. [35]) [36] and some members of the Asgard superphylum, which harbor genes for the synthesis of putative bacterial membrane lipids (see Supplementary Information). The existence of a natural contemporary bacterial counterpart synthesizing archaeal-like membranes might provide weight to the hypothesis that these archaeal groups also synthesize ‘mixed membranes’. Hence, *Ca*. Cloacimonetes, as well as the rest of the FCB group superphylum, appear to be key in our understanding of the ‘lipid divide’. Their membranes may possibly reflect evolutionary remnants of the hypothetical ‘mixed membrane’ of LUCA, or an ancient HGT. In either case, this discovery provides further support for the existence and potential feasibility of ‘mixed membranes’ in natural environments and over a long period in evolutionary history, bridging the lipid divide.

## Supplementary information

Supplementary_Information

Supplementary Tables

## Data Availability

The 16S rRNA gene amplicon reads (raw data) have been deposited in the NCBI Sequence Read Archive (SRA) under BioProject ID PRJNA423140, PRJNA649254–57. The *Ca*. Cloacimonetes MAGs are deposited in IMG under the following IMG accession IDs: 134200 (NIOZ-UU1), 134201 (NIOZ-UU2), 134202 (NIOZ-UU3), 151202 (NIOZ-UU4). The 15 metagenomes (raw data), assembly, and MAGs generated in this study are available under BioProject ID PRJNA649215.
