# Supplementary material for: Bridging the membrane lipid divide: bacteria of the FCB group superphylum have the potential to synthesize archaeal ether lipids
Source: ISME J. 2020 Sep 14;15(1):168–82. doi: 10.1038/s41396-020-00772-2 (PMC7852524; doi:10.1038/s41396-020-00772-2)
Supplement: Supplementary file 1 — Supplementary_Information [file 41396_2020_772_MOESM1_ESM.pdf]

Supplementary Information

Bridging the membrane lipid divide: bacteria of the FCB group superphylum have the potential to synthesize archaeal ether lipids

Laura Villanueva, F. A. Bastiaan von Meijenfeldt, Alexander B. Westbye,
Subhash Yadav, Ellen C. Hopmans, Bas E. Dutilh, and Jaap S. Sinninghe Damsté

Corresponding author: Laura Villanueva
Email: laura.villanueva@nioz.nl

This PDF file includes:

SI Materials and Methods, Results and Discussion
Figures S1-18
References for SI reference citations

Supplementary information includes: Tables S1-S20 are provided as one Excel file, Supplementary File 1 is provided as a PDF file.

SI Materials and Methods, Results and Discussion

Sampling and sample overview. Four research cruises were performed with the R/V Pelagia to the Black Sea as specified in Table S1. Suspended particulate matter (SPM) from 15 depths across the water column (50−2 000 m) was collected at sampling station 2 (N42°53.8’, E30°40.7; 2 107 m depth) during the Phoxy cruise 64PE371 (BS2013) on 9-10 June 2013. DNA was extracted from the BS2013 SPM 0.7 µm pore size glass fiber filters and used to estimate microbial diversity with 16S rRNA gene amplicon sequencing and quantitative PCR (qPCR) as specified below, as well as metagenomic sequencing (see Table S1). Archaeal intact polar lipid data of those SPM filters have been previously reported by Sollai et al. (1). At station 2, water was also collected from 1 000, 1 500, and 1 980 m depth during the NESSC cruise 64PE408 (BS2016) on 31 January-2 February 2016 on Sterivex filter cartridges (Millipore). DNA extracted from the BS2016 samples was used for 16S rRNA gene amplicon sequencing only (Table S2A). Water was also collected from 2 000 m depth during the 64PE444 cruise (BS2018) on 17 August 2018, which was used for attempting enrichment cultures and also to extract DNA which was used for 16S rRNA gene amplicon sequencing and qPCR estimations. Lastly, SPM from 4 depths (500, 1 000, 1 500, and 2 000 m) was collected at sampling station 4 (N42°46.9’, E29°21.1’; 2 100 m depth) during the 64PE418 cruise (BS2017) on 27 March-5 April 2017. DNA was extracted from the BS2017 samples and used for 16S rRNA gene amplicon sequencing. Except for the BS2017 samples (Table S1), total lipids were also extracted to quantify archaeal intact polar lipids as specified below.

Diversity estimates by 16S rRNA gene amplicon sequencing and quantification. Microbial diversity was estimated by 16S rRNA gene amplicon sequencing of the SPM collected during the four cruises (Table S1). In the case of the BS2013 samples, 16S rRNA gene amplicon sequencing was performed initially with 454 GS FLX sequencing as described in Moore et al. (2) and Besseling et al. (3) (Table S2A) and later repeated with Illumina Miseq 2x300 bp as described in van Grinsven et al. (4) to increase sequence resolution and allow for comparison with the 16S rRNA gene sequencing results of later campaigns.

In the BS2013 samples, 16S rRNA gene reads attributed to *Ca.* Cloacimonetes ranged from 5 to 16% of the total bacterial plus archaeal 16S rRNA gene reads between 500 and 2 000 m depth (Table S2). Most sequences belonged to the *Ca.* Cloacimonetes MSBL2 group (13%), while the sequences closely related to *Ca.* Cloacimonetes group MSBL8 represented 3% of the total 16S rRNA gene reads at 2 000 m depth (Fig. 1a, Table S2A). For the BS2013 SPM samples, the relative fraction of reads attributed to *Ca.* Cloacimonetes was similar for the 454 GS FLX sequencing and Illumina MiSeq runs (Table S2B) while the relative fraction of archaeal 16S rRNA gene reads were lower in the Illumina MiSeq libraries. Moreover, the relative fraction of *Ca.* Cloacimonetes reads was similar in the SPM of

BS2013 and BS2016 with percentages ranging from 6 to 14% of total reads between 1 000 and 2 000 m depth (Table S2), while the percentage of *Ca.* Cloacimonetes reads in station 4 of BS2017 was slightly higher (from 7 to 20% from 500 to 2 000 depth). qPCR using primers 515F/806RB was also performed as described in (4) with DNA extracted from SPM from 500, 1 000 and 2 000 m depth obtained during BS2013, SPM from 1 000 and 2 000 m depth collected during BS2018, as well as all SPM samples of station 4 of BS2017. Total 16S rRNA gene abundance estimates were similar from 500 to 2 000 m depth in both station 2 in BS2013, BS2018 and in station 4 in BS2017 with average values of 4 x 10^7 16S rRNA gene copies per liter.

Microscopic detection and characterization of *Ca.* Cloacimonetes at 2 000 m depth. Water samples were also collected at 2 000 m depth during the BS2018 sampling as specified above (see Table S1). Water was collected with Niskin bottles under N_2 overpressure directly into glass pressure bottles acid-washed, autoclaved and also overpressurized with N_2 to minimize exposure to oxygen and keep anoxic conditions. This water was used on board to start enrichments as described in the following section. The presence of *Ca.* Cloacimonetes was assessed in both the original samples and in the enrichments by Catalyzed reporter deposition Fluorescence In Situ Hybridization (CARD-FISH) with the specific HRP-labeled probe Cloa1 5'-GGT TGT GCC CCT TCG GGG G-3', which was designed based on the 16S rRNA gene fragment sequence obtained from the MAG NIOZ-UU1. The CARD-FISH protocol was performed as described earlier ((5) and http://www.environmental-microbiology.de/pdf_files/CARDFISH_2march2013.pdf). To avoid cell loss during cell wall permeabilization, filters were dipped in low-gelling-point agarose (0.2% [wt/vol] in MQ water, dried face up on glass slides at 30°C, and subsequently dehydrated in 96% (vol/vol) ethanol for 1 min. To inhibit endogenous peroxidases water samples from the Black Sea at 2 000 m depth were treated overnight with 0.1% H_2O_2 at room temperature for 2 min. For cell wall permeabilization, filters were incubated in a lysozyme solution (10 mg ml^{-1} in 0.05 M EDTA, 0.1 M Tris-HCl [pH 7.5]) at 37°C for at least 30 min. The sections were washed with MQ water, dehydrated with 96% ethanol, dried at room temperature, and subsequently stored in petri dishes at -20°C until further processing. The optimal stringency for the Cloa1 probe consisted of 55% formamide. A volume of about 30 ml of water collected from the Black Sea at 2 000 m depth as described above was filtered on a 0.22 μm 24 mm diameter polycarbonate filter. A piece of about 1 cm^2 of filter paper was cut and used for CARD-FISH analysis with the Cloa1, EUB388 (general bacteria), and Arc915 (for archaea) HRP-labeled probes (Fig. S1). Visualization was performed on an Axio Imager.M2 Microscope system (Zeiss). Double staining with EUB338-Alexa488 and Cloa1-Alexa555 (in yellow, Fig. S1B) showed the presence of *Ca.* Cloacimonetes cells among the total bacteria. Likewise, the double staining with EUB338-Alexa488 and Arch915-Alexa555 (in yellow, Fig. S1E) showed the presence of archaeal cells among the total bacteria, indicating that the archaeal cells were a minority among the total cells and of smaller size than the *Ca.* Cloacimonetes cells. *Ca.* Cloacimonetes cells were oval to small rods

in shape. The oval shaped cells were 0.8−0.9 µm in diameter whereas small rods were 0.8−9 µm wide and 2−3 µm long.

Genome-centric metagenomics of the Black Sea water column. Assembly and binning of the metagenomes from the SPM samples collected during the BS2013 cruise generated 181 MAGs. NIOZ-UU1−4 fell within the *Ca.* Cloacimonetes phylum (Fig. 1c, Fig. S2). These four MAGs are estimated to be substantial to near complete with low to no detectable contamination (Table S3). Due to difficulties in metagenome assembly of the 16S rRNA gene, only NIOZ-UU1 contained a copy. In support of our shotgun metagenomics and bioinformatic assembly pipeline, we built a maximum likelihood phylogeny of the representative 16S rRNA gene sequences of the amplicon sequencing analysis assigned to the phylum *Ca.* Cloacimonetes, 16S rRNA gene sequences of closely related species obtained from ARB-SILVA (6), the 16S rRNA gene sequence obtained from NIOZ-UU1, and the 16S rRNA gene sequences found in the *Ca.* Cloacimonetes genome placed in Fig. 1c (see also Fig. S2a, Table S4). The tree was inferred with MEGA6 (7) with the generalised time reversible model and gamma distribution, employing 1 000 bootstraps. The comparison of this tree with the concatenated marker gene phylogeny suggests that NIOZ-UU1, 3 and 4 are closely related to MSBL2 group *Ca.* Cloacimonetes genomes (Fig. S2). Mapping shotgun metagenomic sequencing reads back to the cross-assembly revealed that NIOZ-UU3 (MSBL2) was the most abundant *Ca.* Cloacimonetes MAG at 2 000 m depth and showed comparable abundance patterns between MSBL8 and NIOZ-UU2 (Fig. 1).

Predicted metabolism of the Black Sea *Ca.* Cloacimonetes MAGs. Various enzymes related to anaerobic lifestyles were detected in NIOZ-UU1 and NIOZ-UU3 (see Table S5-S8 for functional annotation of NIOZ-UU1 to 4) including ribonucleoside triphosphate reductase, ferredoxin oxidoreductases, and radical S-adenosylmethionine- dependent proteins, which indicated that *Ca.* Cloacimonetes is well adopted to the permanent anoxic conditions of the Black Sea. However, presence of genes related to microaerophilic growth (e.g. superoxide reductase (EC 1.15.1.2), ruberythrin and thioredoxin reductase (EC 1.8.1.9)) in NIOZ-UU1 and NIOZ-UU2 (Table S6) also indicated that the *Ca.* Cloacimonetes found in our samples may also thrive in the suboxic zones of the Black Sea. Alpha-amylase (EC 3.2.1.1) and beta-glycosyl hydrolase, which are involved in the hydrolysis of polysaccharides (cellulose and starch), were detected in NIOZ-UU1 and NIOZ-UU3, supporting their capabilities to obtain energy by hydrolyzing polysaccharides. Furthermore, annotation of NIOZ-UU1 and NIOZ-UU3 also confirmed the presence of genes responsible for the production of ethanol (alcohol dehydrogenase; EC 1.1.1.1).

NIOZ-UU MAGs also harbor genomic indications of a syntrophic lifestyle and of propionate oxidation. Their genomes harbor NiFe dependent hydrogenases, which are known to couple the oxidation of reduced ferredoxin to the production of H_2 during carbohydrate and protein fermentation. NIOZ-UU1 and NIOZ-UU3 also harbor various ferredoxin oxidoreductases, which are primarily

involved in amino acid fermentation. Evidence for syntrophic propionate oxidation have previously been observed for *Ca.* Cloacimonetes *Ca.* Syntrophosphaera thermopropionivorans, and propionate oxidation was speculated to be performed via methylmalonyl CoA but with absence of the complete methylmalonyl CoA pathway genes (8). In the case of the NIOZ-UU MAGs, evidence for this process are the presence of genes coding for the Propionyl-CoA carboxylase beta chain (EC 6.4.1.3) and the Malonyl CoA-acyl carrier protein transacylase (EC 2.3.1.39). Genes involved in glycolysis were also present in NIOZ-UU1 and NIOZ-UU3. However, the complete absence of the electron transfer chain involved in anaerobic respiration indicates that these *Ca.* Cloacimonetes likely obtain their energy through the hydrolysis of polysaccharides, glycolytic pathway and ultimately the fermentation of sugars and amino acids.

The various genes detected in NIOZ-UU1 and NIOZ-UU3 indicated that these *Ca.* Cloacimonetes might be actively involved in the degradation of polysaccharides and amino acids sinking from the upper oxic zones of the Black Sea water column. Furthermore, a complete set of the heterodisulfide reductase (HDR) system was identified in those MAGs. The HDR system serves as an elemental sulfur oxidation enzyme in the cytoplasmic space of bacteria and archaea (9). The HDR system catalyzes the reversible reduction of the disulfide bond (R-S-S-R) coupled with energy conservation (10). The presence of this system in the *Ca.* Cloacimonetes MAGs is expected to be associated with energy generation and conservation by sulfide oxidation in the deeper water column of the Black Sea, where the sulfide concentration can be up to 425 μM (11).

Moreover, a set of genes required for assembly of type IV pili (i.e. Type IV prepilin-like) is encoded in the NIOZ-UU MAGs. Type IV pili can be involved in motility, adherence, DNA uptake, and carrying electric current during direct interspecies electron transfer (DIET) (12), suggesting that the *Ca.* Cloacimonetes detected in the Black Sea may be capable of DIET. Previous studies have also indicated that *Ca.* Cloacimonetes can be involved in cellulose or sugar degradation ((13), among others), which is further supported by the presence of Glucan endo-1,3-beta-glucosidase A1 and Glucosidase YgjK coding genes in NIOZ-UU3. Based on the predicted metabolism of the *Ca.* Cloacimonetes present in the Black Sea water column, we designed a culture enrichment strategy as specified below.

Attempts of enrichment and isolation of *Ca.* Cloacimonetes. We followed different strategies for the enrichment of *Ca.* Cloacimonetes using different growth media by considering the *in situ* environmental parameters of the deep water column of the Black Sea and by extracting information from the four *Ca.* Cloacimonetes MAGs. Six different media were prepared for the selective enrichments. Growth medium 1 (GM1) contained (g L^{-1}, pH 7.0) cellulose (2.0); tryptone (2.0); yeast extract (1.0); $CaCl_2·2H_2O$ (1.0), NaCl (20.0), $MgCl_2·6H_2O$ (3.6), $MgSO_4·7H_2O$ (4.3), KCl (0.5), $Na_2S.9H_2O$ (10 mg). Growth medium 2 was made by 100 times dilution of GM1, while growth medium 3 contained (g L^{-1}, pH 7.0) cellobiose (0.1); KCl (0.55); Na_2SO_4 (2.34); yeast extract (1.0);

$CaCl_2 \cdot 2H_2O$ (2.38), $NaHCO_3$ solution (1 ml of 10% w/v); NaCl (20.0), $MgCl_2 \cdot 6H_2O$ (8.8), ferric citrate solution (5 ml of 0.1% w/v) and sterilized (i.e. autoclaved) Black Sea water (994 ml). Growth medium 4 (GM4) was made by amending GM2 with 10 ml L^{-1} of autoclaved cell lysates. Cell lysates were raised from Black Sea water enriched with GM1. GM5 was made by amending a basal medium containing (L^{-1}) Na_2HPO_4 (5.51 g); KH_2PO_4 (3.4 g); $MgSO_4.7H_2O$ (0.2 g); $CaCl_2.2H_2O$ (0.06 g); $FeSO_4.7H_2O$ (0.5 mg); vitamin solution (2 ml); and trace elements (1 ml) with different amino acids (lysine (4.56 g);leucine (13 mg); isoleucine (13 mg); valine (17 mg); threonine (19 mg); methionine (14 mg); proline (11 mg); arginine (17 mg); histidine (20 mg); phenylalanine (16 mg); cysteine (12 mg); and tryptophan (4 mg). Vitamins and trace elements were prepared as described earlier (14,15). The amino acids and the cofactors were freshly prepared, filter sterilized, and added to the medium after being autoclaved. The headspace was flushed with ultrapure nitrogen. The enrichments were maintained in 120-ml serum vials containing 50 ml of the medium. These anaerobic enrichments were kept at 10°C and 28°C in order to enrich for psychrophilic and mesophilic bacterial members, respectively. Subsamples of 50 µl of the enrichments were pipetted on 0.22 µm 24 mm diameter polycarbonate filters and CARD-FISH was performed as described above. Filters were mounted on microscopic slides with mounting medium containing DAPI and analyzed on an Olympus microscope with 100× magnification. Only the GM4 enrichment showed positive signals with the Cloa1 probe (data not shown). After 10 days of incubation, the enrichments were streaked on the agar media mentioned above and grown in anoxic conditions in anaerobic jars supplemented with Anaerocult® (VWR, The Netherlands). For the isolation and purification of *Ca.* Cloacimonetes from the enrichments, samples were streaked on solid media (1.8% agar). None of the obtained cultures, however, showed a close affiliation with *Ca.* Cloacimonetes based on 16S rRNA gene sequence analysis.

Archaeal lipid biosynthetic genes in *Ca.* Cloacimonetes and beyond. All four *Ca.* Cloacimonetes MAGs contained homologs of the archaeal lipid biosynthetic pathway (see Methods; Fig. S3 for details), including a homolog of the geranylgeranylglyceryl phosphate (GGGP) synthase, co-localized with an homolog of (*S*)-2,3-di-*O*-geranylgeranylglyceryl phosphate (DGGGP) synthase, the two enzymes that mediate the formation of the first and second ether bond in archaeal membrane lipids, respectively (Table S9; Fig. S3). Homology searches show that the two genes are found in other Bacteria and Archaea in the Black Sea water column as well (Fig. S4, Fig. S5; Table S10). Moreover, an extended search in publicly available genomes from other environments and across the tree of life shows that the two genes occur widespread in the FCB group superphylum and related candidate phyla (Fig. 4, Tables S11, S12) although only co-localized in *Ca.* Cloacimonetes (Table S13). Homologs of the two genes were found in representatives from *Ca.* Cloacimonetes, Bacteroidetes, Chlorobi, Calditrichaeota, candidate division Zixibacteria, *Candidatus* Kryptonia, Gemmatimonadetes, Ignavibacteriae, *Candidatus* Edwardsbacteria, *Candidatus* Raymondbacteria, candidate division KSB1

and candidate division TA06, and in one genome of *Candidatus* Parcubacteria of the Candidate Phyla Radiation (16). The two genes are found in *Candidatus* Marinimicrobia and Fibrobacteres as well, but not together in single genomes. Genomes with only one of the genes span an even larger part of the tree of life (Tables S11, S12).

Validation of the metagenomic assembly and co-localization of GGGP and DGGGP synthase *in silico* and experimentally. We thoroughly tested and rejected the possibility that the co-localized bacterial GGGP and archaeal DGGGP synthase coding genes in the *Ca.* Cloacimonetes MAGs could have been introduced by a methodological error. First, chimeras could have been produced during cross-assembly, i.e. sequencing reads that are not derived from the same species in the original sample could have been merged into scaffolds. Second, scaffolds could have been binned erroneously into MAGs, e.g. because of relaxed binning parameters or because binning signals (coverage across samples and TNF) are very similar between species. To address the first hypothesis, we plotted coverage along the full length of the scaffolds of interest in their deepest covered samples, and found the scaffolds evenly covered (Fig. S6), the remaining small-scale peaks in part reflecting biases introduced during the Truseq-nano library preparation. Importantly, the GGGP and DGGGP synthase ORFs are co-localized on a single assembled scaffold in NIOZ-UU1, NIOZ-UU3, and NIOZ-UU4, and read coverage across them is continuous (Fig. S6a and Fig. S6c-d). Whereas GGGP and DGGGP synthase coding genes are not directly co-localized on the same scaffold in NIOZ-UU2, one read-pair bridges the two scaffolds and an alignment between the two scaffolds and GGGP synthases in the three other MAGs shows the two genes to be concatenated as well in NIOZ-UU2 (data not shown). Moreover, the scaffold of interest in NIOZ-UU3 carries both the archaeal lipid biosynthesis genes and a bacterial (node ID: UID2495) marker gene, PriA (primosomal protein N′) from the TIGR00595 family, which is present single-copy in the MAG, and again, connected to the lipid genes through even coverage (Fig. S6c). We, therefore, conclude that the scaffolds are not chimeras. To address the second hypothesis of erroneous binning, we compared the average read coverage profiles across samples for the scaffolds of interest. Importantly, for the four MAGs, the scaffolds of interest show comparable coverage profiles across samples as the full MAGs (Fig. S7). Moreover, each of the four MAGs has a unique coverage profile (data not shown). Thus, we have no reason to suspect that the scaffolds of interest are archaeal sequences that were erroneously binned with *Ca.* Cloacimonetes scaffolds. Furthermore, subsequent database searches identified the presence of both the GGGP and DGGGP synthase encoding genes in a range of other FCB group and related candidate phyla genomes as well, further supporting the existence of a bacteria synthesizing both bacterial and archaeal membrane lipids (Tables S11, S12).

We additionally verified our binning-analysis experimentally by PCR and sequencing: DNA extracted from the 1 000 and 2 000 m depth SPM obtained during BS2013 were PCR-amplified using primers designed to span the GGGP and DGGGP synthases, polyprenyl synthase and the marker gene

PriA in the specific scaffold of NIOZ-UU3 (Table S9). The resultant amplicon only from the 2 000 m depth SPM sample was cloned and sequenced, confirming our assembly (data not shown).

Quantification of GGGP and DGGGP synthase coding gene and gene expression. In order to evaluate if the *Ca.* Cloacimonetes 'archaeal'-like membrane lipid biosynthetic pathway is functional, we determined the transcriptional activity of the GGGP, DGGGP, and polyprenyl synthase genes by a RT-PCR approach. The positive expression of those genes was confirmed in the SPM 1 000 and 2 000 m depth samples of BS2013 (Fig. S8). No amplification was detected when a negative control of the reverse transcription reaction (RNA extract without reverse transcriptase) was included as DNA template of the PCR reaction (Fig. S8). Amplified fragments were further sequenced to confirm the products (data not shown).

In addition, we performed a qPCR approach to quantify the NIOZ-UU3 GGGP and DGGGP coding genes both at the DNA and RNA level and through the vertical profile of SPM from 50 to 2000 m depth of the BS2013 campaign (15 depths). The *Ca.* Cloacimonetes GGGP synthase coding gene was detected from 250 m depth downwards with a maximum of 1.7×10^7 copies per liter at 2 000 m depth (qPCR efficiency = 80%, R2 = 0.997; Fig. S9). Gene transcripts of the *Ca.* Cloacimonetes GGGP synthase coding gene were also detected from 250 m depth downwards with a minimum value at this depth (2.7×10^2 copies L^{-1}; detection limit qPCR assay estimated to be 15 copies L^{-1}), which increased with depth to a maximum of 1.8×10^4 copies L^{-1} at 2 000 m depth. These results point to an increasing number of *Ca.* Cloacimonetes GGGP synthase genes with depth, in agreement with the estimation of *Ca.* Cloacimonetes 16S rRNA copies L^{-1} based on 16S rRNA gene amplicon sequencing and the 16S rRNA gene qPCR assay as estimated above. Gene expression was detectable but low. It is likely that gene expression of this gene may be affected by the sampling procedures we are applying, as the SPM samples do not get fixed until they are retrieved on the deck of the ship, after they have gone through decompression from deep waters (i.e. 2 000 m depth) to surface in a short period of time (approximately 30 min). These factors have been seen to significantly affect the gene expression profile of deep sea samples (17). Similarly, the *Ca.* Cloacimonetes DGGGP synthase abundance and gene expression was evaluated by qPCR, with qPCR efficiency too low for an accurate quantification. However, we estimated the *Ca.* Cloacimonetes DGGGP synthase gene to be detectable from 250 m depth downwards and with an increasing abundance reaching maximum values at 2 000 m depth (Fig. S10A). *Ca.* Cloacimonetes DGGGP synthase gene transcripts were also detected from 250 m downwards, which was supported by a positive qPCR signal and correct melting behaviour in the melting curve (Fig. S10B).

Biochemical verification of the encoded archaeal-like lipid biosynthesis proteins. In order to confirm the enzymatic function of the putative GGGP synthases annotated in the four *Ca.* Cloacimonetes MAGs, the amplified GGGP synthase open reading frame (ORF) from NIOZ-UU3

(Table S14) was expressed from a T7-promoter in plasmid pABW4 in *E. coli* BL21(DE3), and the 6His tagged protein was purified by Ni-NTA affinity chromatography (see Methods; Table S15). Purity was verified using 12% TGX™ precast gels (Bio-Rad), stained with Bio-Safe™ Coomassie stain (Bio-Rad). The purified recombinant *Ca.* Cloacimonetes GGGP synthase (Fig. S11) used in the enzymatic assay below was identified based on the predicted size. Note that three larger bands were also present (Fig. S11B) and tentatively identified as multimers of the purified GGGP synthase, which is in line with the observations of Peterhoff et al. (18), who reported that all group I GGGP synthases are dimers, while group II GGGP synthases enzymes are either dimers or hexamers.

The enzymatic activity of the protein was tested in an assay with geranylgeranyl diphosphate (GGPP, 20 carbons) and either G1P or G3P as substrates to further test the stereo-selectivity of the enzyme, performed in duplicate. Samples were analyzed by UPLC-HRMS (see Methods), and GGGP formation (detected as GGGP ammonium adduct $[GGGP+NH_4]^+$ (Fig. 2a, Fig. S12) and GGGP-sodium adduct $[GGGP+Na]^+$, not shown) was observed both in the presence of G1P and G3P (Fig. 2a, Fig. S12), consistent with previous studies of archaeal GGGP synthases (19–21). The identification of GGGP was confirmed by MS^2 fragmentation analysis of the GGGP produced (Fig. S13a) and furthermore had a retention time and MS^2 fragmentation pattern consistent with the structural analogue C_{18}-lyso phosphatidylglycerolphosphate (1-O-octadecyl-2-hydroxy-*sn*-glycero-3-phosphate; Avanti Polar Lipids, Cat no. 857228). No GGGP formation was detected in the absence of enzyme, GGPP or glycerol-phosphate. For GGGP-positive samples, some variation in the amount of GGGP was observed and the use of G1P appeared to result in increased GGGP levels compared to G3P (Fig. S12), consistent with the reported preference of archaeal GGGP synthases for G1P over G3P (18–21).

To test whether the GGGP and DGGGP synthases encoded by the *Ca.* Cloacimonetes MAGs could support the formation of archaeal-like lipids in a bacterium, we co-expressed NIOZ-UU3 GGGP and DGGGP synthases (encoded on plasmids pABW1, -2 and -3) in *E. coli* C43(DE3), a strain optimized for membrane protein production (22) that contained plasmid pMS148 (23). Plasmid pMS148 encodes GGPP synthase (*crtE*) and G1PDH (*araM*), enzymes that produce G1P and GGPP, respectively, the likely biosynthetic substrate for the *Ca.* Cloacimonetes GGGP synthase. For analysis, lipids were extracted from cells and analyzed by Ultra High Pressure Liquid Chromatography – High Resolution Mass Spectrometry (UHPLC-HRMS) for the formation of archaeal lipid intermediates (see Methods).

Cells expressing both GGGP and DGGGP synthases produced significant amounts of phosphatidylglycerol archaeol with 8 double bonds or unsaturations (Fig. 2b, Fig. S14; PG-unsat(8)-archaeol, i.e. an octaunsaturated (8 double bonds or unsaturations) archaeol, *n*-2,3-diphytanylglycerol diether with isoprenoid chains of 20 carbons, with a phosphatidylglycerol head group, also known as phosphatidylglycerol digeranylgeranylglyceryl phosphate), the expected intermediate in the biosynthesis of archaeal membrane lipids in the absence of a specific geranylgeranyl reductase in *E. coli* (24,25). The identity of this compound was verified using MS^2 fragmentation analysis and the

interpretation of the fragmentation spectrum of PG-unsat(8)-archaeol was based on Yoshinaga et al. (26) (Fig. S13b). No PG-unsat(8)-archaeol was detected in cells lacking either GGGP or DGGGP synthase, or in cells lacking the upstream enzymes GGPP synthase and G1PDH (encoded on pMS148) (Fig. S14).

Other lipid biosynthetic genes in *Ca.* Cloacimonetes. We subsequently focused on genes of the lipid biosynthesis pathways other than GGGP and DGGGP synthase encoding genes present in the four Black Sea MAGs affiliated with *Ca.* Cloacimonetes. The gene coding for the glycerol-3-phosphate dehydrogenase (G3PDH, *gps* gene) catalyzing the formation of G3P was detected in three of the MAGs (NIOZ-UU1, 3, and 4; Table S16). In addition to genes for GGGP and DGGGP synthase, other genes required for the synthesis of isoprenoidal archaeal lipids were also detected in the *Ca.* Cloacimonetes MAGs, including the genes for a complete bacterial isoprenoid MEP/DOXP pathway (Table S17), genes coding for acetyl-CoA C-acetyltransferase and hydroxymethylglutaryl-CoA synthase of the Mevalonate pathway (see Table S17, Fig. S3), and two polyprenyl synthases (Table S17). In addition, several genes of the bacterial fatty acid pathway were detected in the MAGs (Table S18), including the acyltransferases responsible for the esterification of G3P, as well as genes coding for enzymes involved in downstream reactions (Table S16, Fig. S3). The complete annotation of NIOZ-UU1 to 4 is found in Tables S5–S8.

A phylogenetic analysis of the polyprenyl synthases indicated the presence of close relatives in other *Ca.* Cloacimonetes genomes, and they were classified as either short-chain or geranylgeranyl diphosphate (GGPP) synthases and medium-chain length prenyltransferases based on their sequence (18) (Fig. S15a). Finally, the four *Ca.* Cloacimonetes MAGs also included a putative digeranylgeranylglycerophospholipid reductase (identified based on homology with experimentally verified geranyl reductase, (23)) that is also closely related to homologs of the *Ca.* Cloacimonetes and FCB group superphylum genomes. These homologs are closely related to putative digeranylgeranylglycerophospholipid reductases of archaeal genomes of the Euryarchaeota (Fig. S15b). Both these two maximum likelihood phylogenetic analyses were performed with PHYML v3.0 (27) using the model indicated by ProtTest 2.4 (28) (LG model plus gamma distribution and invariant site, LG+G+I). Sequences were aligned with MUSCLE (29). Alignments were trimmed with Gblocks 0.91b (30) using relaxed parameters and manually curated.

Potential sources of archaeal membrane lipids in the Black Sea deep water column. Archaeal lipid diversity and abundance was estimated in the SPM samples collected from station 4 (500, 1 000, 1 500 and 2 000 m depth) during BS2017. The diversity of IPL-derived CLs detected in station 4 in 2017 was similar to that observed in station 2 in 2013 (see (1)) with predominance of GDGT-1 and 2 as well as archaeol (Table S19). The total archaeal IPLs abundance increased from 2.5 to 25 ng per liter from 500 to 2 000 m depth (Table S19). We next assessed whether the archaeal IPLs observed in

the water column could be attributed to the living archaeal cells. All calculations are available in Supplementary File 1. Absolute abundances of total 16S rRNA gene copies per liter were determined by qPCR as described above (Table S2) in the samples from which IPLs were measured. Cells per liter for a given taxon at each depth was estimated by multiplying the fraction of total 16S rRNA gene amplicon sequencing reads attributed to that taxon by the total 16S rRNA gene copies per liter estimated by qPCR and dividing by the expected 16S rRNA gene copy number in the genome (Supplementary File 1). For the archaeal groups we assumed one 16S rRNA gene per genome, and for *Ca.* Cloacimonetes two, as the genome of "*Ca.* Cloacimonas acidaminovorans" str. Evry contains two copies (https://rrndb.umms.med.umich.edu/, (31)). We estimated the IPLs abundances that the observed archaeal cells could theoretically produce at each depth based on the estimated abundances of archaeal cells per group, their estimated cell size based on literature, and different models for lipid production per cell surface area (Supplementary File 1). We took uncertainty in measurements for both lipid production and cell size estimates into account by including the most extreme cases reported in literature, arriving at a range for the amount of IPLs that the archaeal population could produce. The maximum of this range represents an ideal case scenario, where all archaea in the water column are at maximum known size and produce the maximum reported number of membrane lipid molecules. Lipid abundance estimates were based on the proposed 0.86–1.85 femtograms (fg) of archaeal lipids per cell for rods sized 0.5–0.9 µm length × 0.2 µm width (32), 1 fg per cell for 0.8 × 0.5 µm rods (33), and 0.25 fg per cell for 0.5 × 0.15 µm rods (34). Cell size estimates for the different archaeal groups were: Thermoplasmatales (rods 0.5–3 µm length × 0.2–0.5 µm width (35)), ANME-1 (cells within aggregates 1.2 µm length × 0.3–0.4 µm width (36)), Bathyarchaeota (spherical cells 0.4–0.5 µm size (37)), and DPANN Woesearchaeota (spherical cells between 400 and 500 nm diameter inferred based on the diameter of the DPANN *Nanoarchaeum equitans,* 400 nm (38) and the 500 nm diameter of the DPANN ARMAN Nanoarchaea (39)). For the remaining archaeal cells (referred to as "archaea, others" in Table S2) we took a range between 0.25 and 5 femtograms of archaeal lipids per cell.

We arrive at a predicted IPLs concentration of <6.5 ng per liter based on the archaeal population (Supplementary File 1). This represents a striking offset at 1 000, 1 500 and 2 000 m depth ranging from 2 to 5 fold between observed archaeal IPLs and predicted IPLs. This means that even the most ideal situation, where all Archaea in the water column are of maximum known size and produce the maximum reported number of membrane lipid molecules cannot explain the observed amount of archaeal IPLs at deeper depths. An explanation for this mismatch could be that suspended IPLs are preserved for a long time in the anoxic waters of the Black Sea. In deep anoxic sediments, preservation as fossils of archaeal IPLs with stable glycosidic bonds has been observed (e.g. (40)). However, this seems an unlikely explanation as the IPLs reported here are found in the water column, which is expected to have a much higher degradation rate than sediments. Moreover, most of the archaeal IPLs detected in the Black Sea do not contain the stable glycosidic-based polar head groups but rather the more labile headgroups phosphatidylglycerol, phosphatidylserine, or

phosphatidylethanolamine (1). We, therefore, argue that the mismatch provides evidence for the production of archaeal-like ether-linked isoprenoid membrane lipids by the highly abundant *Ca.* Cloacimonetes bacteria at these depths, which are 2–4 times more abundant than the total archaeal population.

Archaeal lipid biosynthetic genes in *Ca.* Cloacimonetes and other bacteria. The GGGP synthases of the Black Sea *Ca.* Cloacimonetes MAGs have close homologs in other recently released *Ca.* Cloacimonetes genome sequences and in members of the FCB group superphylum and related candidate phyla (Fig. 4, Fig. S16, Tables S11, S12). Our phylogeny shows a clear separation between group I and group II GGGP synthases in line with earlier findings (41), with the *Ca.* Cloacimonetes sequences falling within group II (Fig. 4, Fig. S16). Crenarchaeota contain the closest archaeal relatives to the detected GGGP synthases (Fig. 4, Fig. S16), however the phylogeny of this enzyme is inconclusive regarding the origin of the GGGP synthase homologs in the FCB group superphylum. The extended presence and close phylogenetic associations of the GGGP synthase homologs in this superphylum and related candidate phyla strongly supports the presence of this enzyme in Bacteria at least before radiation of the FCB group. Thus, this GGGP synthase could be a 'remnant' of the 'mixed membrane' stage after LUCA and before the diversification of Bacteria. Alternatively, the GGGP synthase could have been transferred after the bacterial membrane origin from Archaea to the ancestor of the FCB group and related bacterial phyla, in line with earlier suggestions (41).

Like GGGP synthase, the DGGGP synthase genes detected in the four *Ca.* Cloacimonetes MAGs (Table S9) also have close homologs in other *Ca.* Cloacimonetes, FCB group superphylum, and recently released candidate phyla genomes (Fig. 4, Fig. S17, Tables S11, S12). The DGGGP synthases of *Ca.* Cloacimonetes are closely related to TACK group genomes, in particular Crenarchaeota and *Candidatus* Korarchaeota (Fig. 4, Fig. S17). The topology of the phylogeny is similar to that of GGGP synthase with respect to sharing the TACK group as sister clade and branching of bacterial clades, suggesting that the two genes share a similar evolutionary history. Interestingly, many genomes from the class Flavobacteriia within the phylum Bacteroidetes contain the gene in double copy (Table S11), suggestive of a duplication within the phylum. Sequence identity between the Bacteroidetes cluster and other bacterial/archaeal sequences is low (Fig. S18), which might be an indication for a divergent activity of the gene in the phylum. DGGGP synthases belong to a superfamily of UbiA prenyltransferases (42) including prenyltransferases of the ChlG/BchG (i.e. chlorophyll *a* synthase ChlG from *Synechosystis* sp. strain PCC6803, accession number BAA10281) and UbiA/COQ2 prenyltransferases for the biosynthesis of ubiquinone (AAK40480.1_ubiA-1_*Sulfolobus solfataricus*; CAA96321.1_COQ2 *Saccharomyces cerevisiae*; AAC43134.1_4-hydroxybenzoate octaprenyltransferase UbiA *Escherichia coli*), therefore we included some of these sequences to possibly elucidate the LUCA or HGT origin of the DGGGP synthase gene. However, the placement of these 'outgroups' close to the base of the archaeal tree does not unequivocally solve the

question of the origin of the gene; the phylogenetic signal in DGGGP synthase is insufficient to identify the archaeal species tree, wherever a root is placed.

Screening of membrane lipid biosynthetic genes in the Asgard archaea. Previous studies have reported that two uncultured archaeal groups, the Euryarchaeota Marine Group II and Lokiarchaeota of the Asgard superphylum, contain archaeal lipid biosynthesis genes alongside bacterial-like fatty acid and ester-bond formation genes, but apparently they lack the gene coding for glycerol 1-P-dehydrogenase (G1PDH), suggesting that they are not able to synthesize the typical archaeal-like lipids with G1P stereochemistry (43). This observation is interesting as these genomes also have the genes required to produce G3P, making it possible that they synthesize archaeal-like lipids with G3P stereochemistry. The study by Caforio et al. (19) suggests that G1P could be synthesized in the absence of the typical G1PDH, thus archaea of the Marine Group II Euryarchaeota and some of the Asgard archaea might still synthesize G1P-archaeal like lipids. They might also synthesize fatty acid-based G3P bacterial-like lipids as they have some of the genes for fatty acid synthesis, G3P synthesis and acyltransferases for the esterification of the fatty acids to the G3P backbone. We further screened currently available Asgard archaea MAGs for the presence of the genes of the archaeal and bacterial lipid biosynthetic pathways (Table S20). In contrast to the Lokiarchaheota MAG CR4, Asgard archaea MAGs of the *Ca.* Heimdallarchaeota, Odinoarchaeota and Thorarchaeota do harbor a G1PDH coding gene homolog. In addition, several of the *Ca.* Heimdallarchaeota MAGs (Table S20) also harbor a homolog of the PlsY acyltransferase, and both the *Ca.* Heimdallarchaeota MAGS LC2 and LC3 (Table S20) also have homologs of the bacterial PlsC acyltransferase as observed for the Lokiarchaeota CR4 MAG. The lack of G1PDH in Lokiarchaeota and potentially of G1P-archaeal like lipids in Asgard archaea is very appealing for supporting the eukaryogenesis scenario with eukaryotes originating from within the Asgard archaea (44). However, the presence of G1PDH in other Asgard archaea as seen here weakens this hypothesis. Also, the absence of a gene from incomplete MAGs reconstructed from environmental samples is not a warranty that the gene is actually missing from the genome. However, we also confirmed the absence of the G1PDH homolog (i.e. blastp with the G1PDH of *Ca.* Odinarchaeota archaeon LCB_4 as query sequence) in the complete genome of an archaeon of the Lokiarchaeota that has been recently obtained from an enrichment culture (45). We therefore consider this further support that Asgard Lokiarchaeota either do not synthesize G1-based archaeal-like membrane lipids or they use a novel and unexpected alternative pathway to synthesize G1P as observed in the study of Caforio et al. (19) for the bacterium *E. coli*. Regardless of this genetic evidence, there is still no experimental evidence of the formation of a 'mixed membrane', neither in the Asgard archaea nor in the Marine Group II Euryarchaeota.

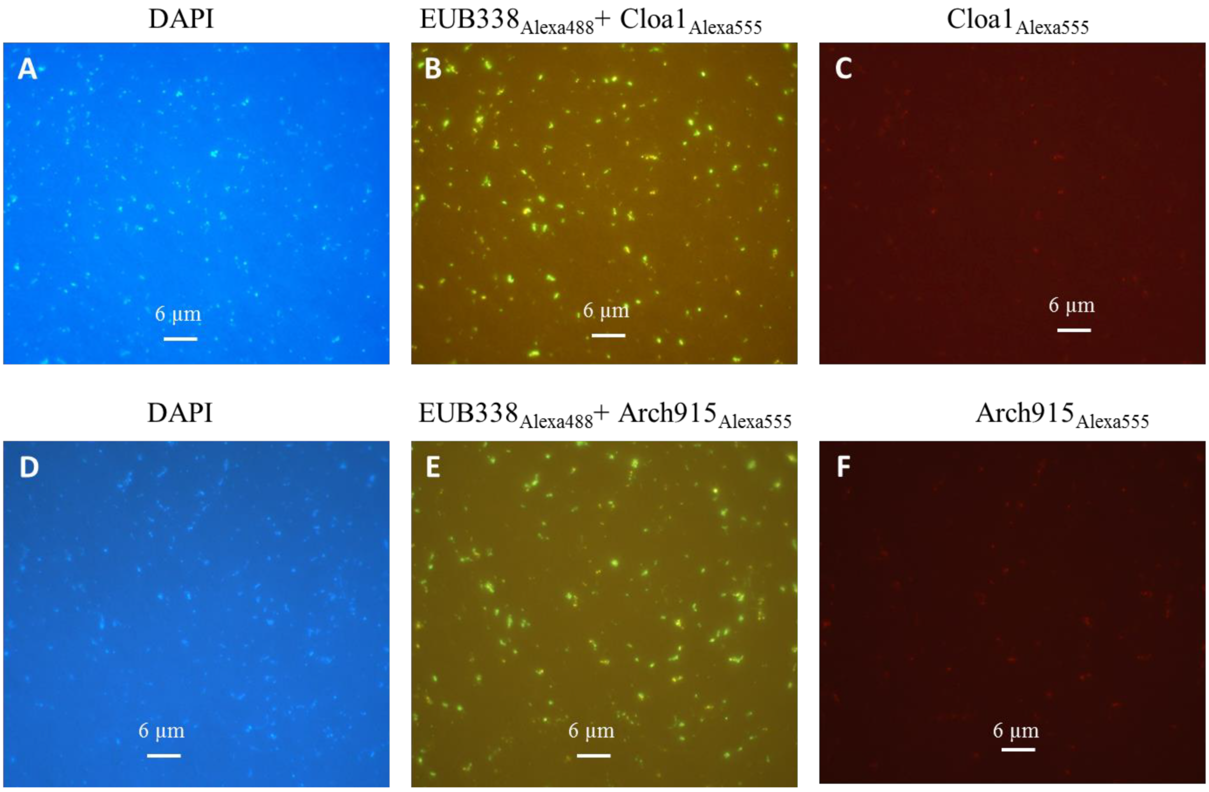

Fig. S1. Epifluorescence microscope images of *Ca.* Cloacimonetes cells hybridized by Catalyzed reporter deposition Fluorescence In Situ Hybridization (CARD-FISH) with either DAPI (**A,D**) or with the specific HRP-labeled probes Cloa1 for *Ca.* Cloacimonetes (**B,C**), EUB388 for general bacteria (**B,E**), and Arch915 for archaea (**E, F**) of Black Sea water collected at 2 000 m depth. Images were obtained with ×63 magnification.

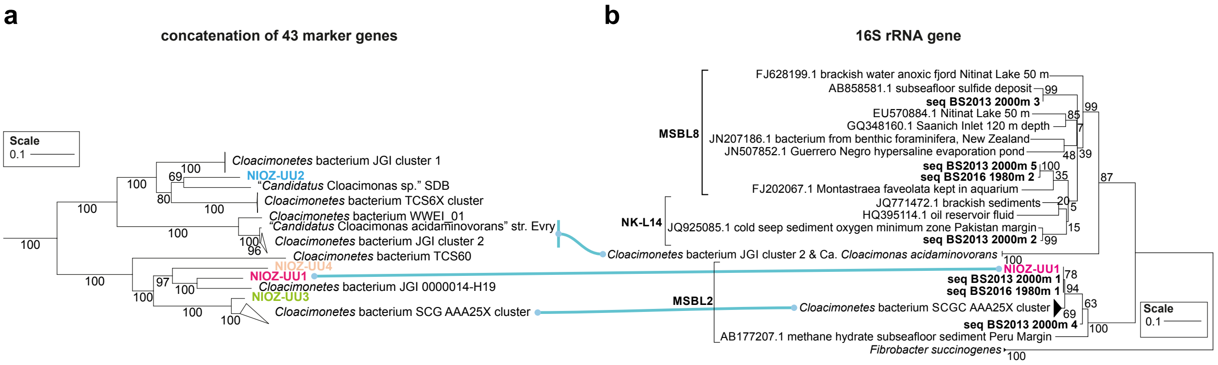

Fig. S2. Phylogenetic affiliation of the *Ca.* Cloacimonetes MAGs. **a**, Maximum likelihood phylogenetic tree based on 43 concatenated marker genes indicating the position of the *Ca.* Cloacimonetes MAGs with respect to other *Ca.* Cloacimonetes genome sequences available in databases. See methods for details. **b**, 16S rRNA gene phylogenetic tree inferred by using the maximum likelihood method based on the generalised time reversible model, applying the neighbor-joining method with a discrete gamma distribution plus invariable sites. The analysis involved a total of 1661 positions. Evolutionary analyses were conducted in MEGA6 (7). Correspondence of the sequences between the two trees is indicated with blue lines. 16S rRNA sequences from NIOZ-UU1, seq_BS2013_2000m_1, and seq_BS2016_1980m_1 are identical. For a description of the clustered sequences, see Table S4. Scale bars in (**a**) and (**b**) indicate mean number of amino acid and nucleotide substitutions per site, respectively.

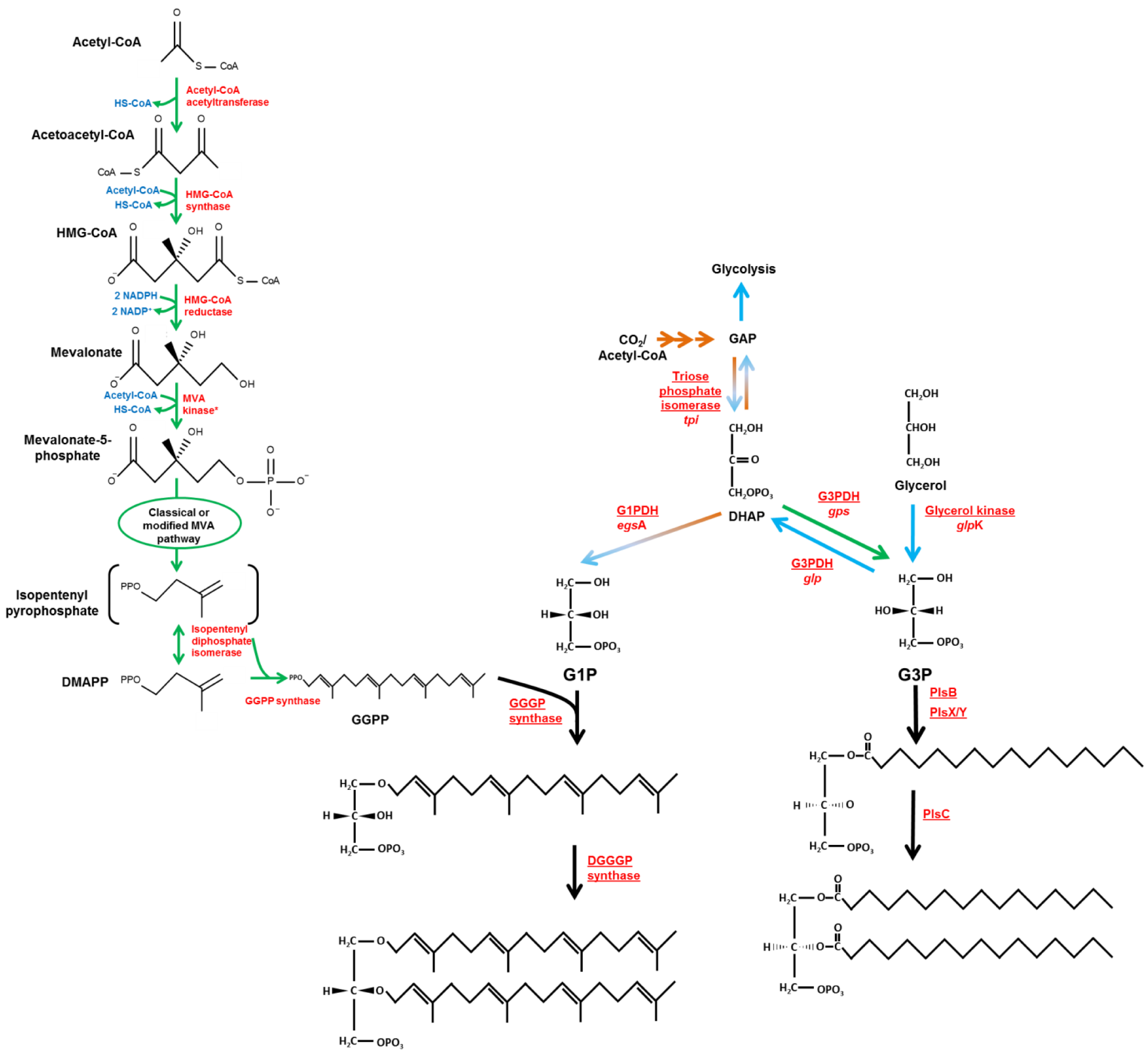

Fig. S3. Scheme of the archaeal membrane lipid biosynthetic pathway listing the main genes and enzymes involved.

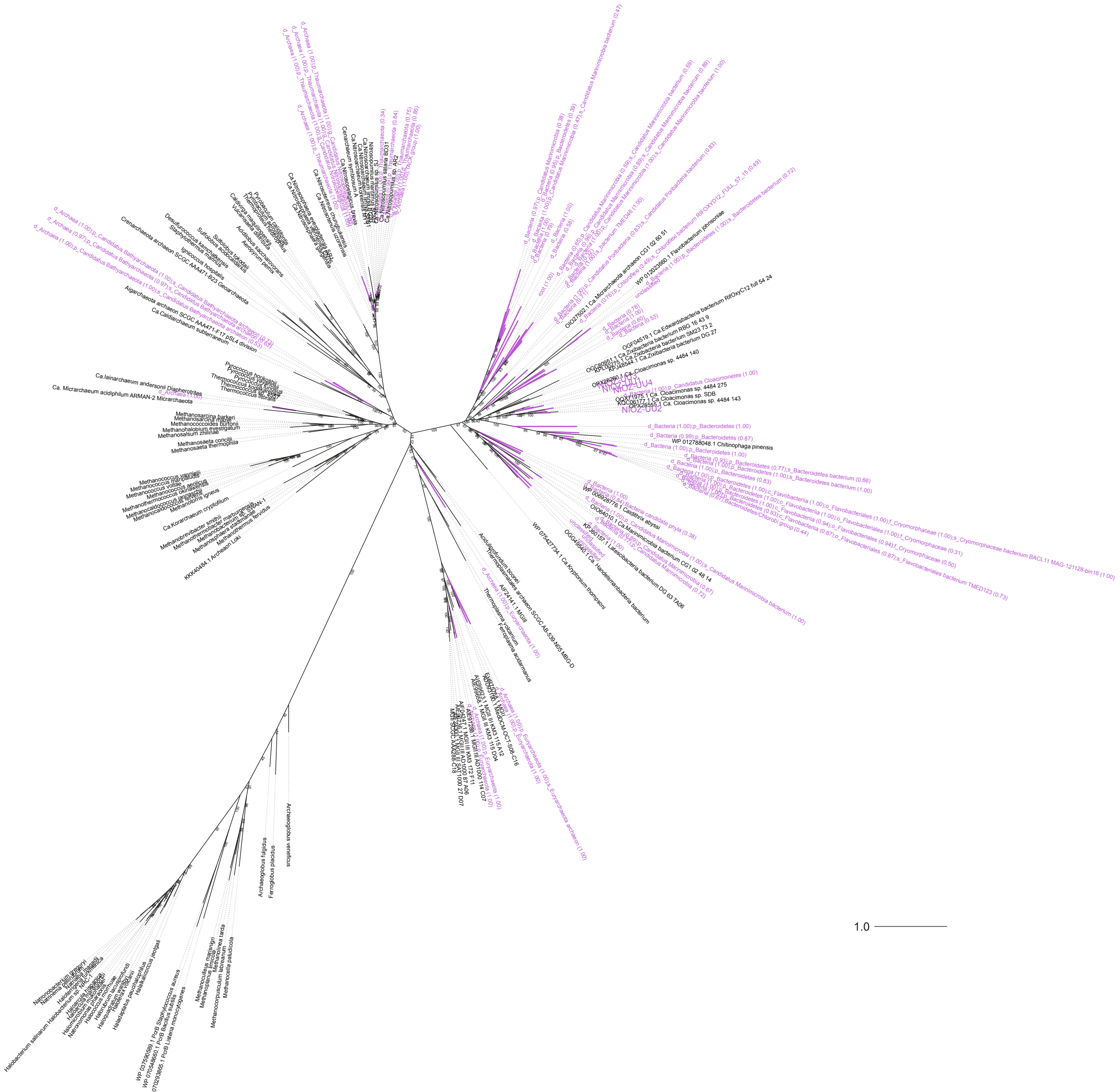

Fig. S4. Maximum likelihood tree of GGGP synthases found in the Black Sea assembly (purple) and reference sequences (black). *Ca.* Cloacimonetes reference sequences are based on a blastp search against nr. Branch support is based on 1 000 ultrafast bootstraps. Scale bar represents mean number of substitutions per site. Taxonomic classification of the sequences from the assembly are based on taxonomic classification of the scaffolds on which they were found with Contig Annotation Tool (CAT). Only classifications at official taxonomic ranks are shown, unless intermediate ranks are informative (e.g. ‘candidate division TA06’). Numbers between brackets indicate the fraction of bit-score support for that classification (see (46)). Note that taxonomic classifications that have low bit-score support and/or are based on few ORFs (see Table S10 for full CAT results) are speculative.

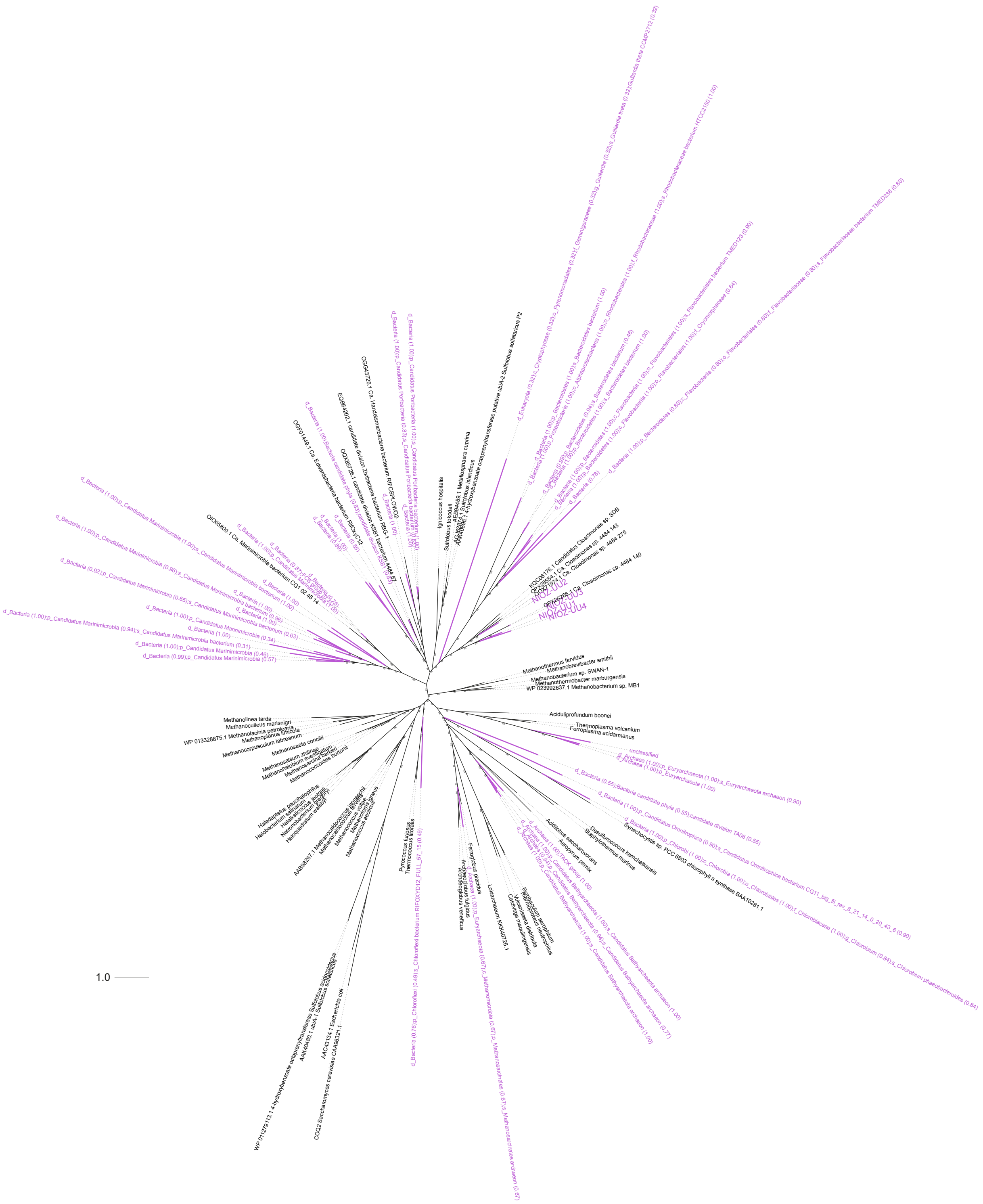

Fig. S5. Maximum likelihood tree of DGGGP synthases found in the Black Sea assembly (purple) and reference sequences (black). *Ca.* Cloacimonetes reference sequences are based on a blastp search against nr. Branch support is based on 1 000 ultrafast bootstraps. Scale bar represents mean number of substitutions per site. Taxonomic classification of the sequences from the assembly are based on taxonomic classification of the scaffolds on which they were found with Contig Annotation Tool (CAT). Only classifications at official taxonomic ranks are shown, unless intermediate ranks are informative (e.g. 'TACK group' if there is no lower rank classification). Numbers between brackets indicate the fraction of bit-score support for that classification (see (46)). Note that taxonomic classifications that have low bit-score support and/or are based on few ORFs (see Table S10 for full CAT results) are speculative. The eukaryotic scaffold classification is tentative because it is ultimately based on a single ORF out of 30 that was classified as *Guillardia theta* (see Table S10).

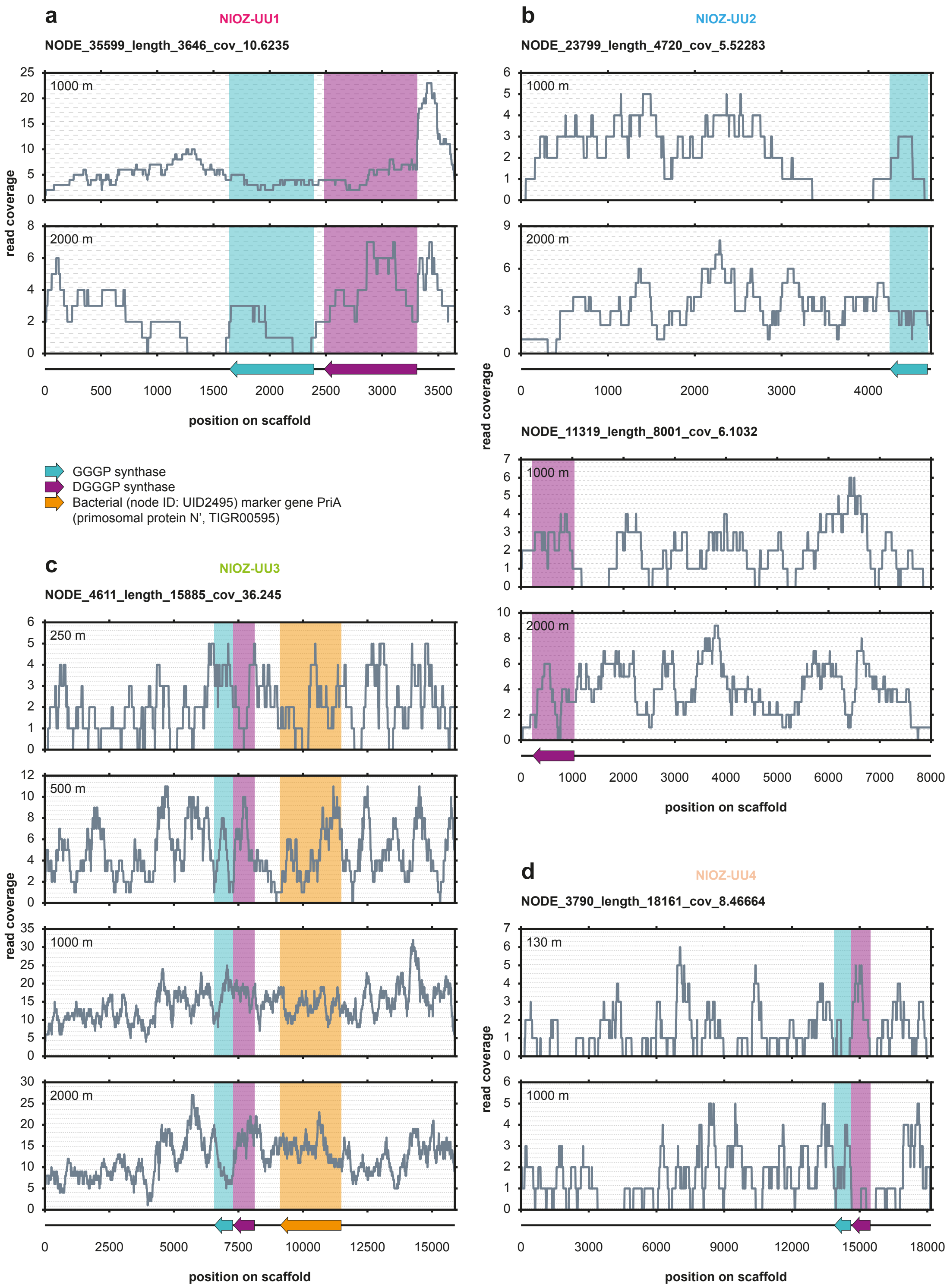

Fig. S6. Coverage across *Ca.* Cloacimonetes scaffolds containing GGGP and DGGGP synthase. **a-d**, Panels showing the position of the genes on the scaffolds, together with read coverage per nucleobase, as generated with samtools mpileup. Coverage is only shown for samples in which average read coverage is considerable (see horizontal dashed lines in Fig. S7). **b**, In NIOZ-UU2 the genes are located on different scaffolds. **c**, The location of a bacterial marker gene on the same scaffold as the two archaeal homologs in NIOZ-UU3 is shown.

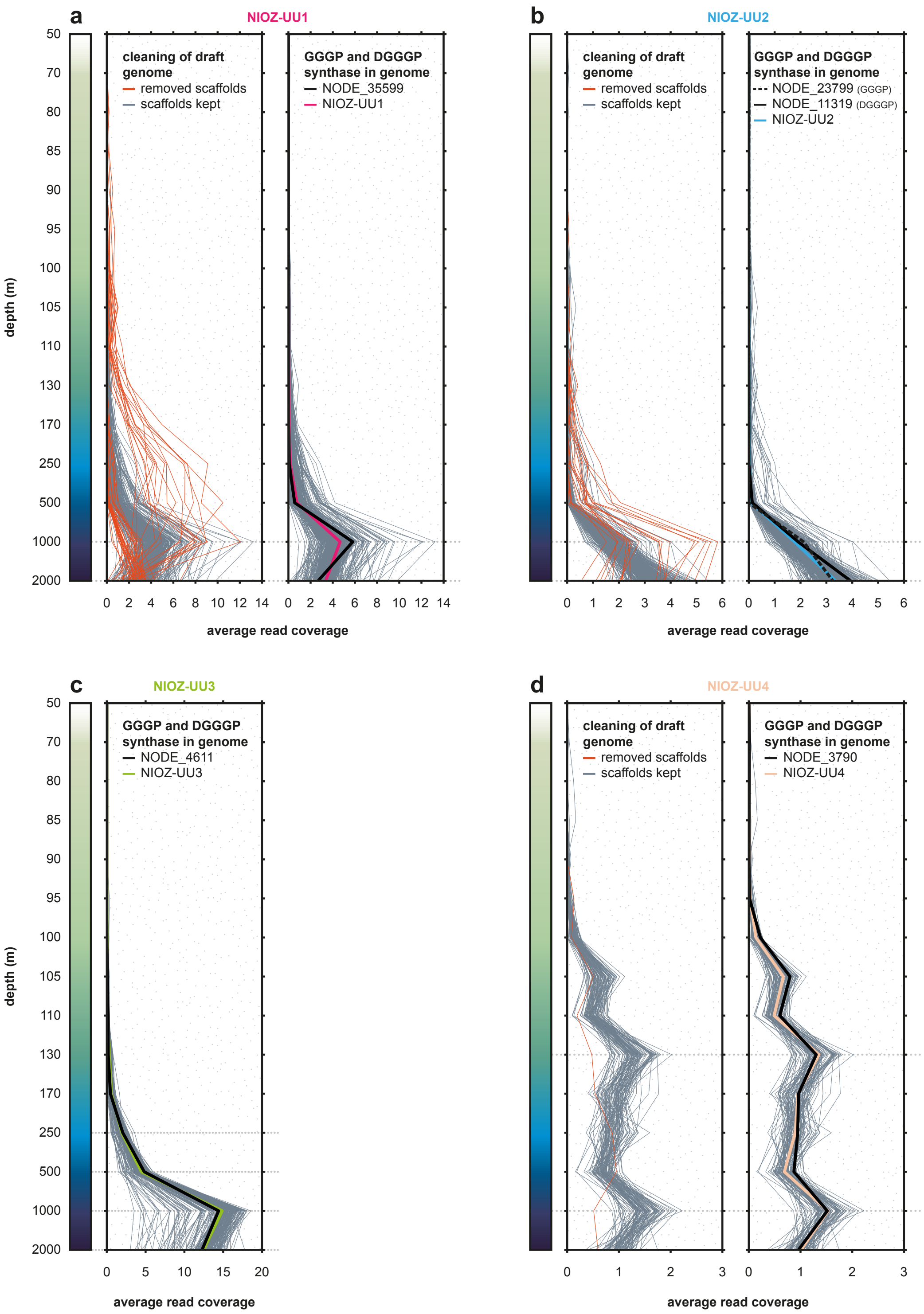

Fig. S7. Average read coverage (depth of mapped reads per nucleobase) for all the scaffolds in the four MAGs. Left panels in (**a,b,d**) show removed scaffolds due to manual cleaning. Since the coverage profile of NIOZ-UU3 looks clean (**c**), no scaffolds were removed. The right panel in (**a-d**) shows average read coverage of the scaffolds in the cleaned MAGs, with the scaffolds that contain GGGP and DGGGP synthase genes highlighted (2 in NIOZ-UU2). Colored lines depict average read coverage of the cleaned MAGs, see Methods for details. Horizontal dashed lines indicate depths at which read coverage across the scaffolds containing GGGP and DGGGP synthase is plotted in Fig. S6.

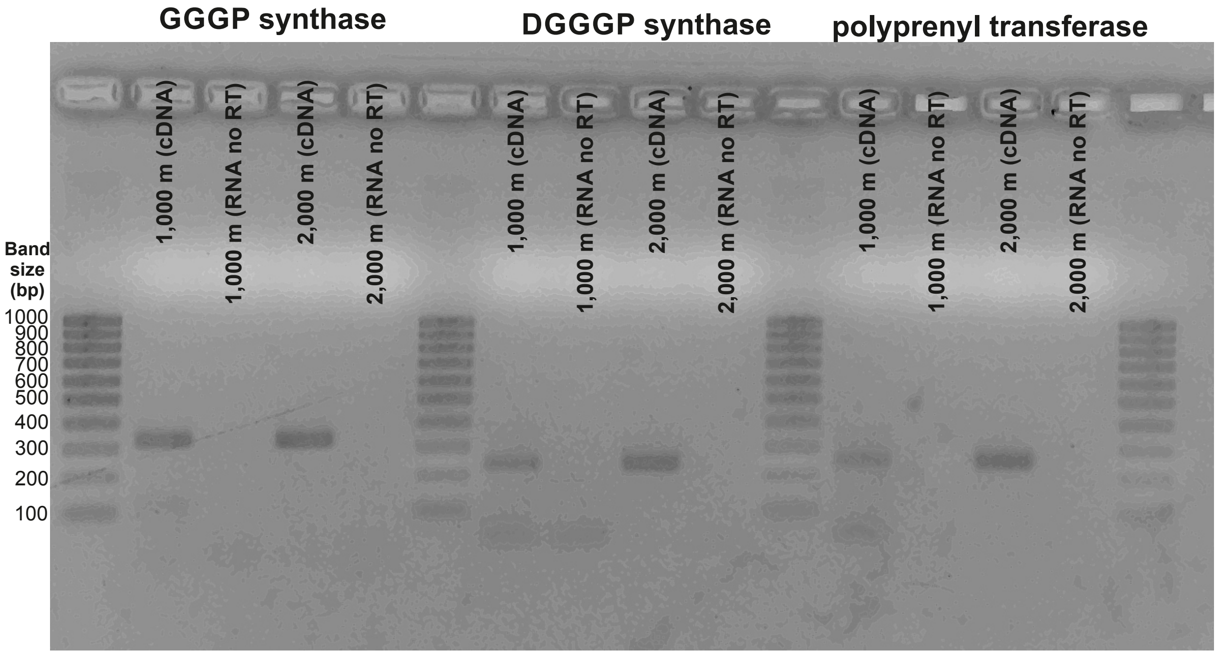

Fig. S8. Assessment of gene expression of the putative GGGP, DGGGP and polyprenyl transferases of NIOZ-UU3 in the 1 000 and 2 000 m depth Black Sea samples. Band indicates positive amplification with the primers listed in Table S17. no RT: indicates negative control of RNA extract with absence of reverse transcriptase. cDNA: PCR using complementary DNA generated by reverse transcription. Amplicons were further sequenced for verification (data not shown).

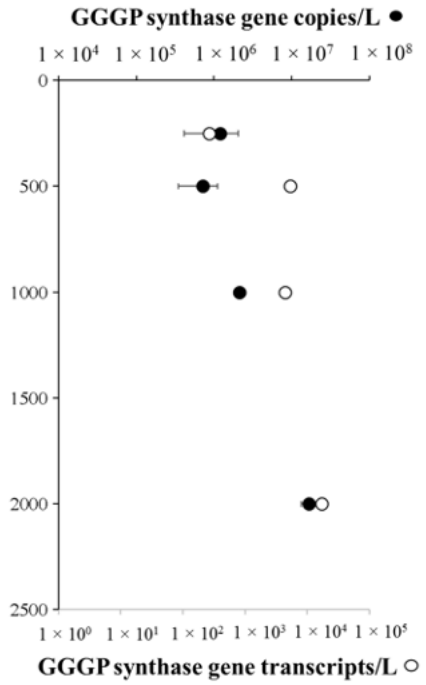

Fig. S9. *Ca.* Cloacimonetes GGGP synthase gene (black circles) and gene transcripts (white circles) copies per liter estimated by qPCR in the BS2013 campaign SPM samples.

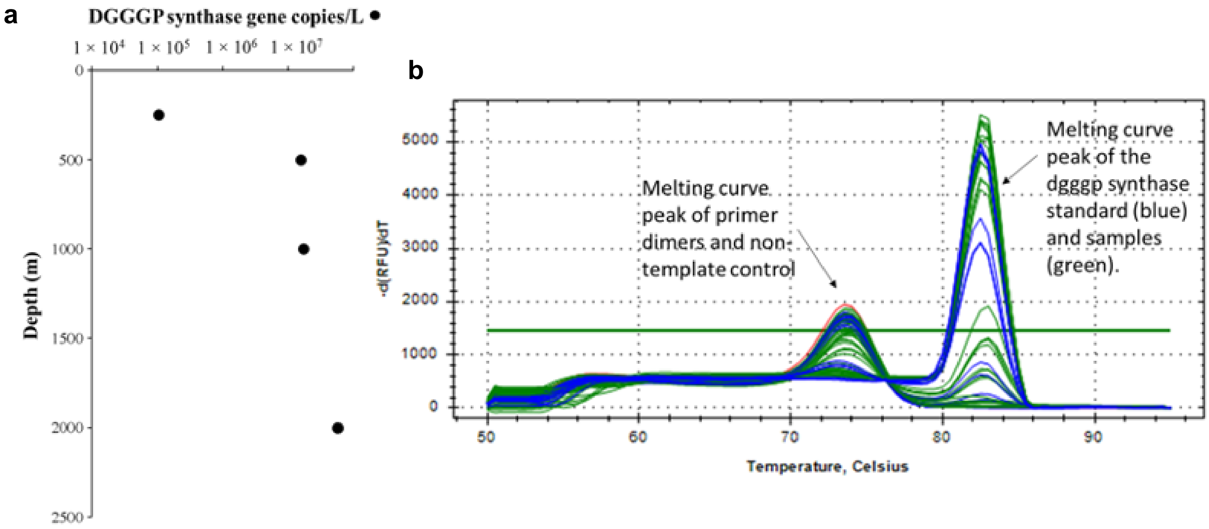

Fig. S10. *Ca.* Cloacimonetes DGGGP synthase coding gene and gene expression quantification. **a**, *Ca.* Cloacimonetes DGGGP synthase gene (black circles) copies per liter estimated by qPCR in the BS2013 campaign SPM samples. Note that the abundance is not accurate as explained in the text. **b**, Melting curve of the *Ca.* Cloacimonetes DGGGP synthase gene and gene transcript quantification, indicating that the melting curve behavior of the standard (PCR amplicon of the *Ca.* Cloacimonetes DGGGP synthase gene fragment) is identical to that found in gene and gene transcript estimations in SPM extracts from 250 m downwards.

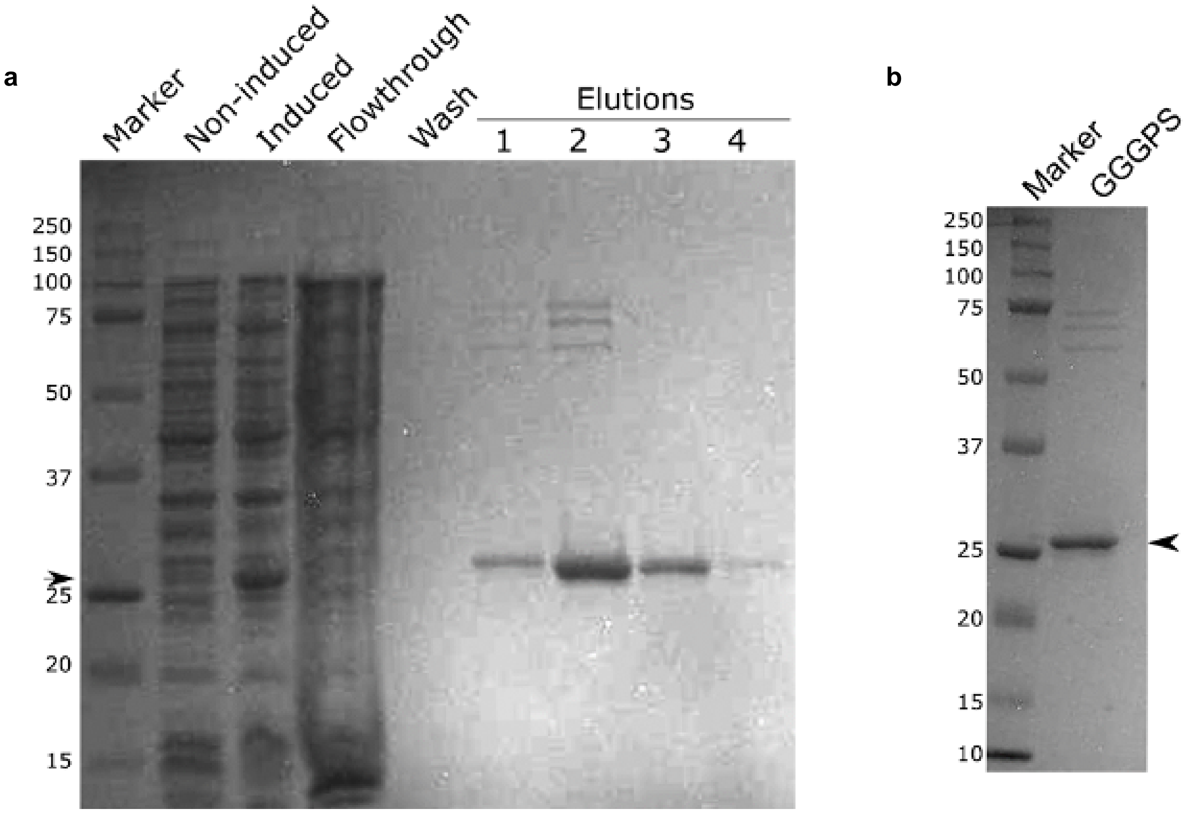

Fig. S11. SDS-PAGE gel images of the purified recombinant *Ca.* Cloacimonetes GGGP synthase. **a**, Gel indicating the protein extract in the different purification phases. Elution 2 was used in the enzymatic assay. **b**, Re-run of the protein extract obtained in Elution 2 with less material. Arrow points to predicted size (see text for further details).

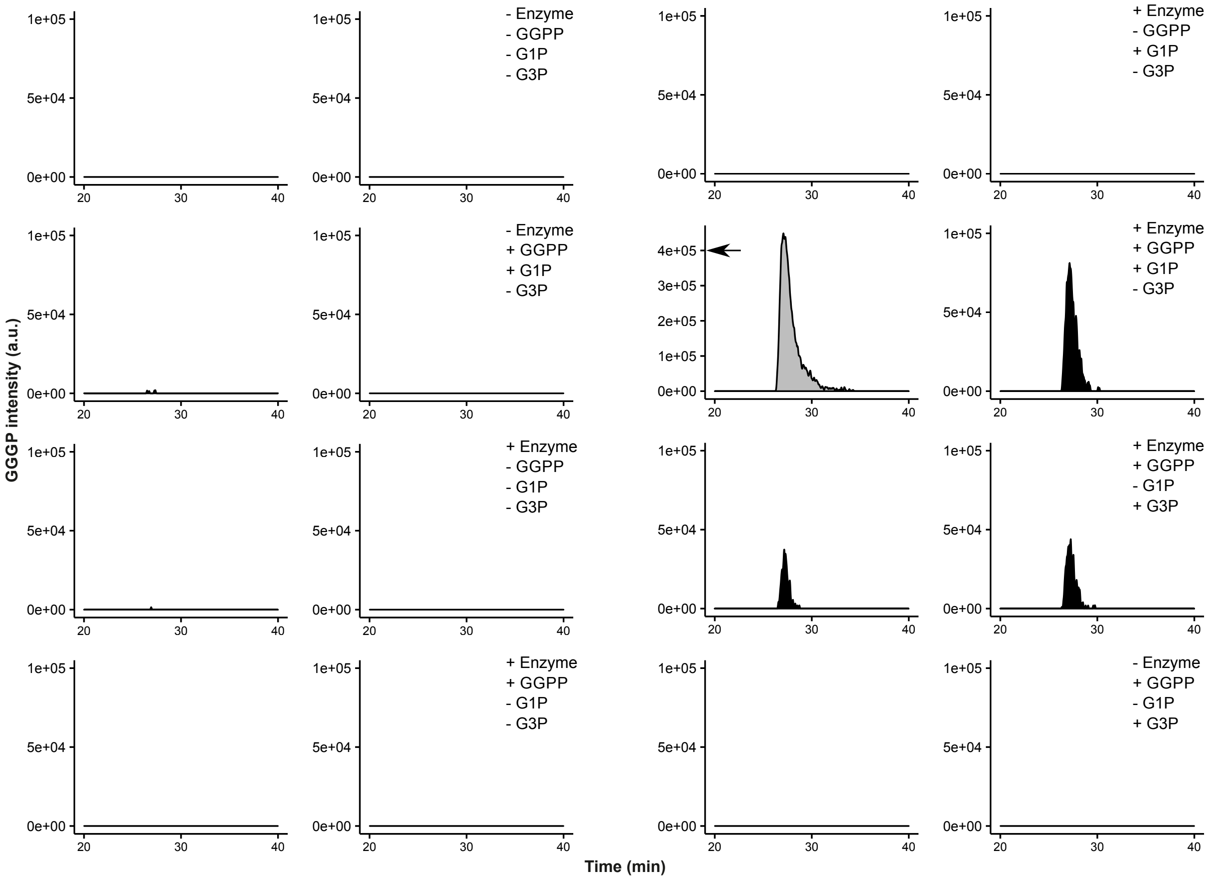

Fig. S12. GGGP production *in vitro*. Extracted ion chromatogram within 3 parts per million (ppm) mass accuracy, of $[GGGP+NH_4]^+$ (*m/z* 462.298) showing the retention time in minutes *vs* the GGGP intensity in arbitrary units, a.u. Results with inclusion or absence of purified enzyme, geranylgeranyl-diphosphate, glycerol-1-phosphate (G1P) or glycerol-3-phosphate (G3P) in reaction assay as indicated. Two replicate enzyme assays (performed different days) are indicated. Note that one sample (grey fill) is plotted with a 5-fold increased max. y-scale value (arrow), to allow visual comparison to the other samples.

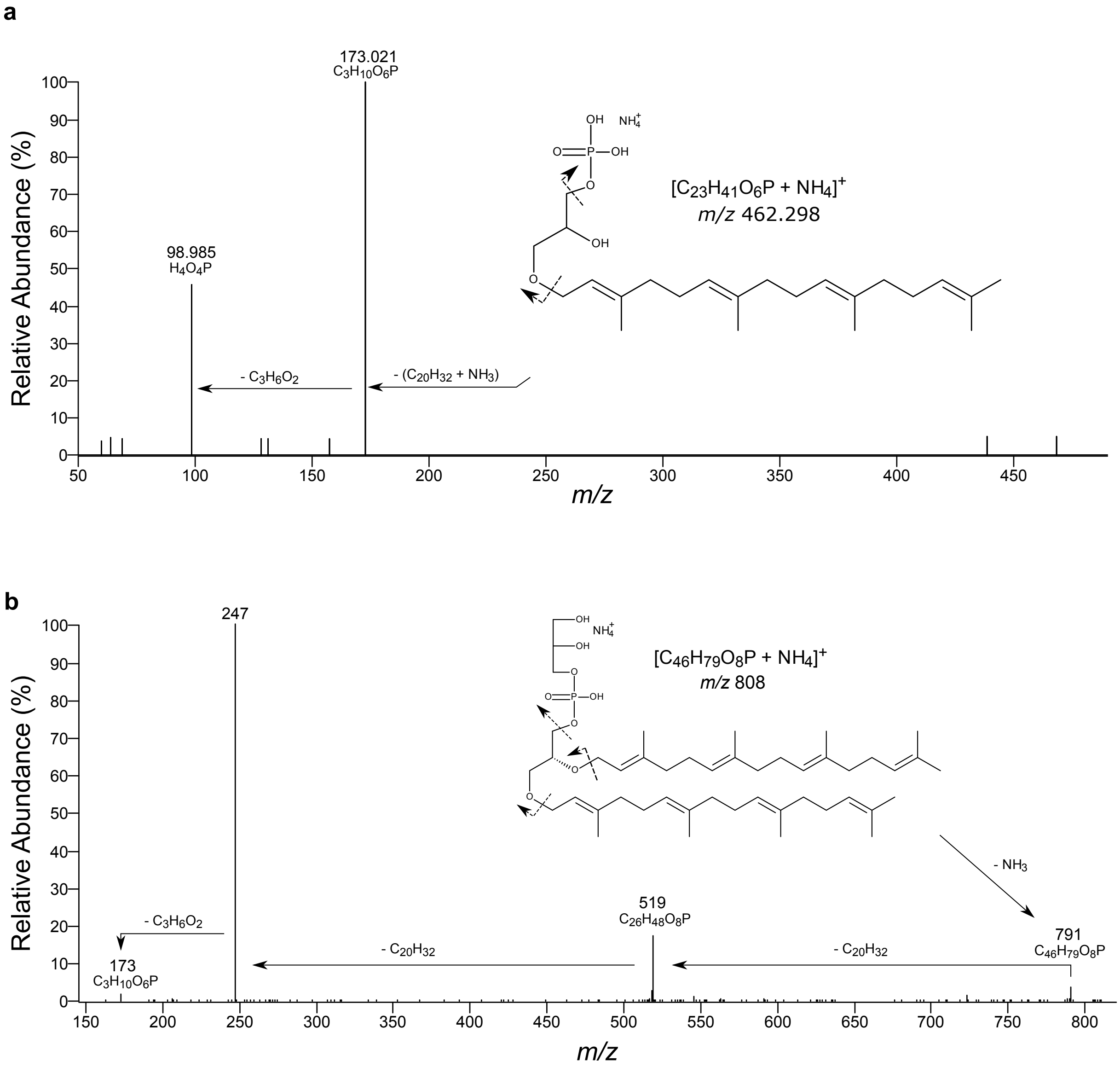

Fig. S13. Representative MS^2 fragmentation spectra of the GGGP produced in vitro and the phosphatidylglycerol-archaeol with 8 double bonds produced by the recombinant *E. coli* strain. **a**, Geranylgeranylglyceryl phosphate (GGGP) produced *in vitro*, obtained by HRMS using a quadrupole orbitrap hybride MS. The MS^2 fragmentation spectrum showed a loss of the geranylgeranyl carbon chain ($-C_{20}H_{32} + NH_3$) generating a fragment at *m/z* 173.021 representing the phosphatidylglycerol ($C_3H_{10}O_6P$). Subsequent loss of the glycerol moiety ($-C_3H_6O_2$) results in a fragment at *m/z* 98.985 representing the remaining phosphatidic acid moiety (PO_4H_4). **b**, Phosphatidylglycerol-archaeol with 8 double bonds or unsaturations (PG-unsat(8)-archaeol) produced by recombinant *E. coli* C43(DE3) obtained by ion trap MS, as described in (supplementary) Materials and Methods. The fragmentation mass spectrum of PG-unsat(8)-archaeol) shows an initial loss of NH_3 resulting in formation of the $[M+H]^+$ at *m/z* 791. Subsequent losses of the 2 geranylgeranyl moieties ($2 \times -C_{20}H_{32}$) results in the formation of fragments at *m/z* 519 and 247, with the latter representing the glycerol backbone with phosphatidylglycerol headgroup. Loss of a glycerol moiety results in the formation of the fragment at *m/z* 173 representing phosphatidylglycerol.

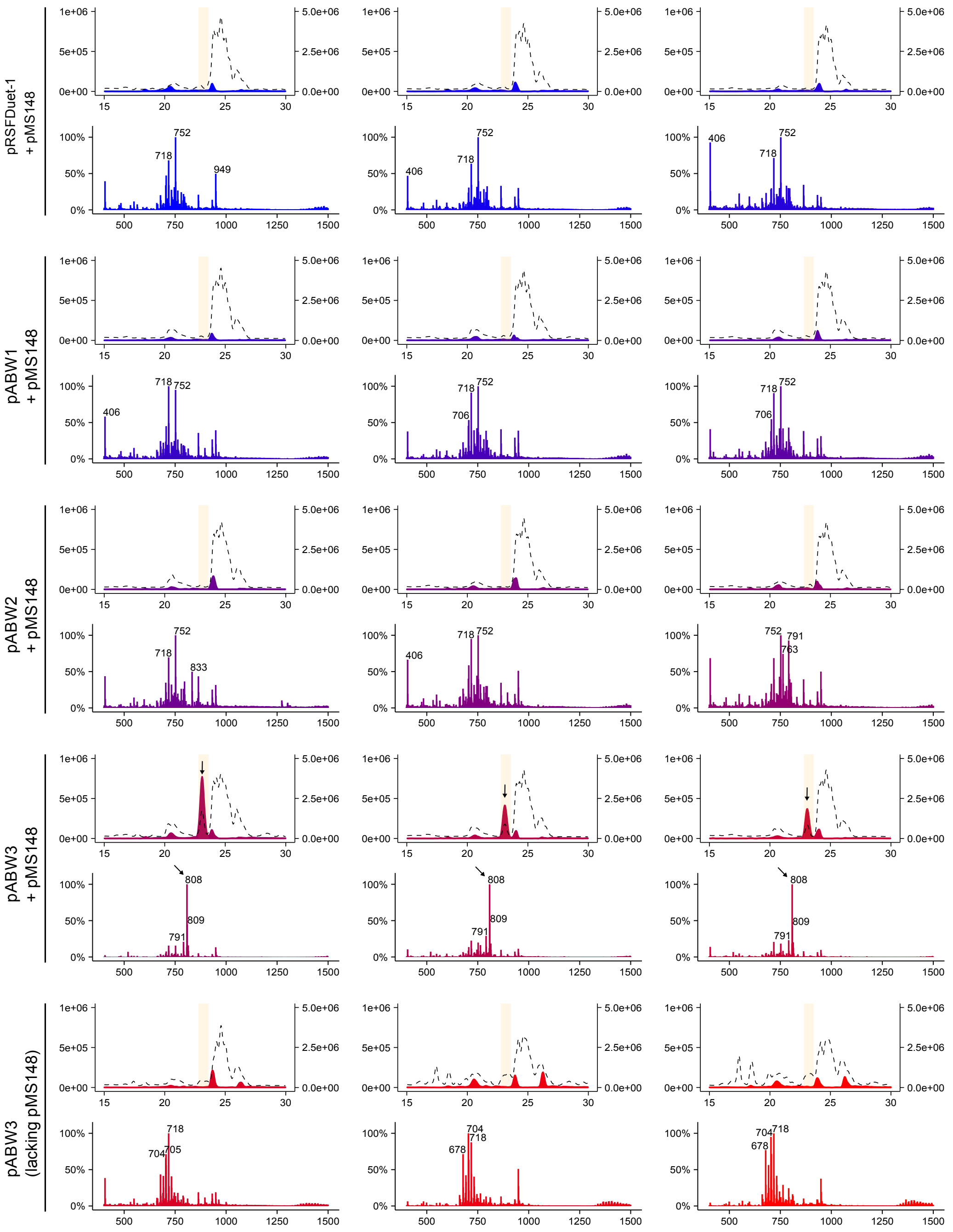

Fig. S14. PG-unsat(8)-archaeol production in recombinant *E. coli* C43(DE3). Upper panel of each subfigure shows the extracted ion chromatogram (± 0.5 mass units) of [PG-unsat(8)-archaeol+H]$^+$ (*m/z* 808; filled colored area, left y-axis [PG-unsat(8)-archaeol+H]$^+$, in arbitrary units, a.u.), and the base peak intensity in a.u. (right y-axis, dotted line) *vs* the retention time in minutes (x-axis). Orange box highlights the retention time where PG-unsat(8)-archaeol is detected (in the positive samples) and used for summed MS^1 analysis. The lower panel shows the average MS^1 spectrum for the region spanning the PG-unsat(8)-archaeol retention time (22.8 to 23.3 min) is shown with the three most intense *m/z* indicated (x-axis, m/z; y-axis, relative abundance in percentage). The plasmids harbored by *E. coli* C43(DE3) (see Table S15) are indicated and arrows indicate formation of PG-unsat(8)-archaeol. The analysis results of three biological replicates are shown.

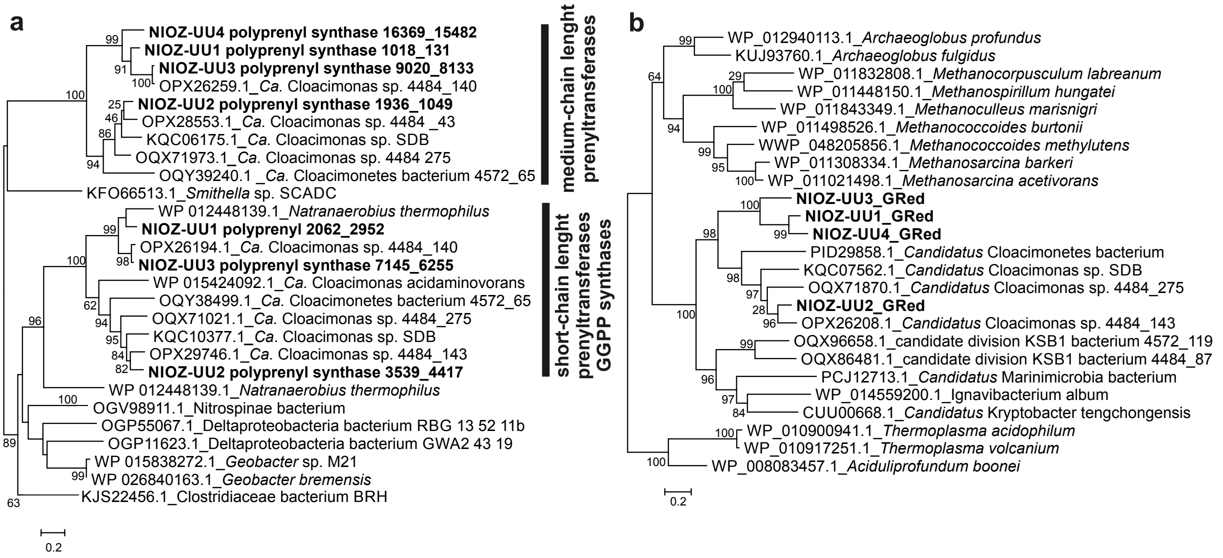

Fig. S15. Phylogeny of the polyprenyl transferases and digeranylgeranylglycerophospholipid reductases detected in the *Ca.* Cloacimonetes MAGs. **a**, The two putative polyprenyl transferases, and **b**, putative digeranylgeranylglycerophospholipid reductases and closest relatives. Scale bar represents mean number of substitutions per site. Branch support was calculated with the approximate likelihood ratio test (aLRT) and values ≥50% are indicated on the branches.

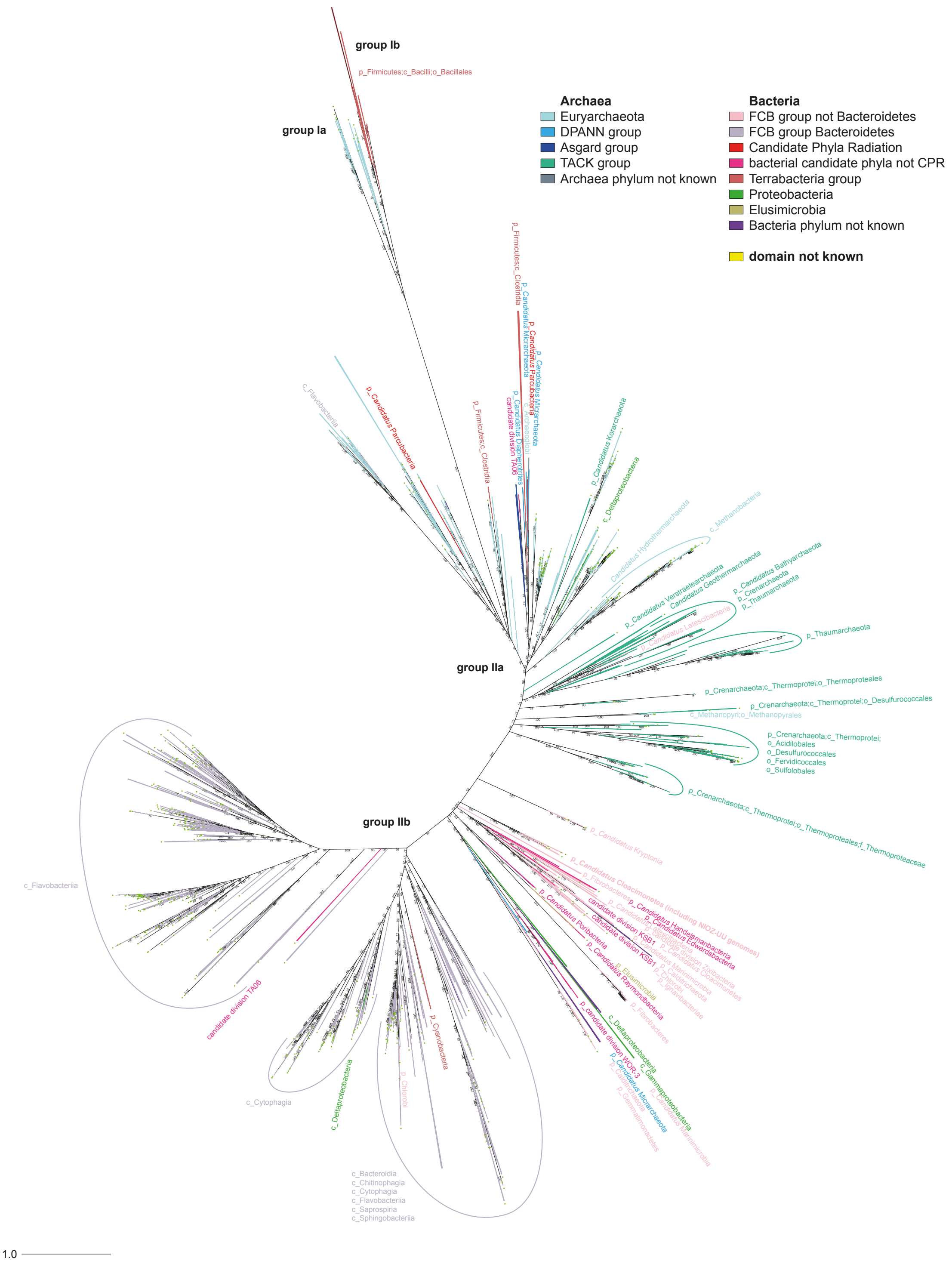

Fig. S16. Consensus tree of GGGP synthases across the tree of life. The tree is based on 1,068 unique sequences representing 1,389 genomes from cultures and environmental samples. Bacterial clades are labeled as a certain group if a large fraction of sequences in the clade shows a consistent annotation. Interesting or aberrant placements are labeled as well, as are close archaeal sister groups to the bacterial part of the tree. Groups *sensu* Peterhoff et al. (18). Branch support is based on 1 000 ultrafast bootstraps. Scale bar represents mean number of substitutions per site. Archaeal or bacterial 'phylum not known': phylum is not known but the genome is annotated on a lower level, or sequence represents multiple groups. 'Domain not known': genomes for which no lineage was found on the PATRIC servers. Green dots: DGGGP synthase is also found in the same genome, or in at least one of the genomes if the branch represents multiple genomes.

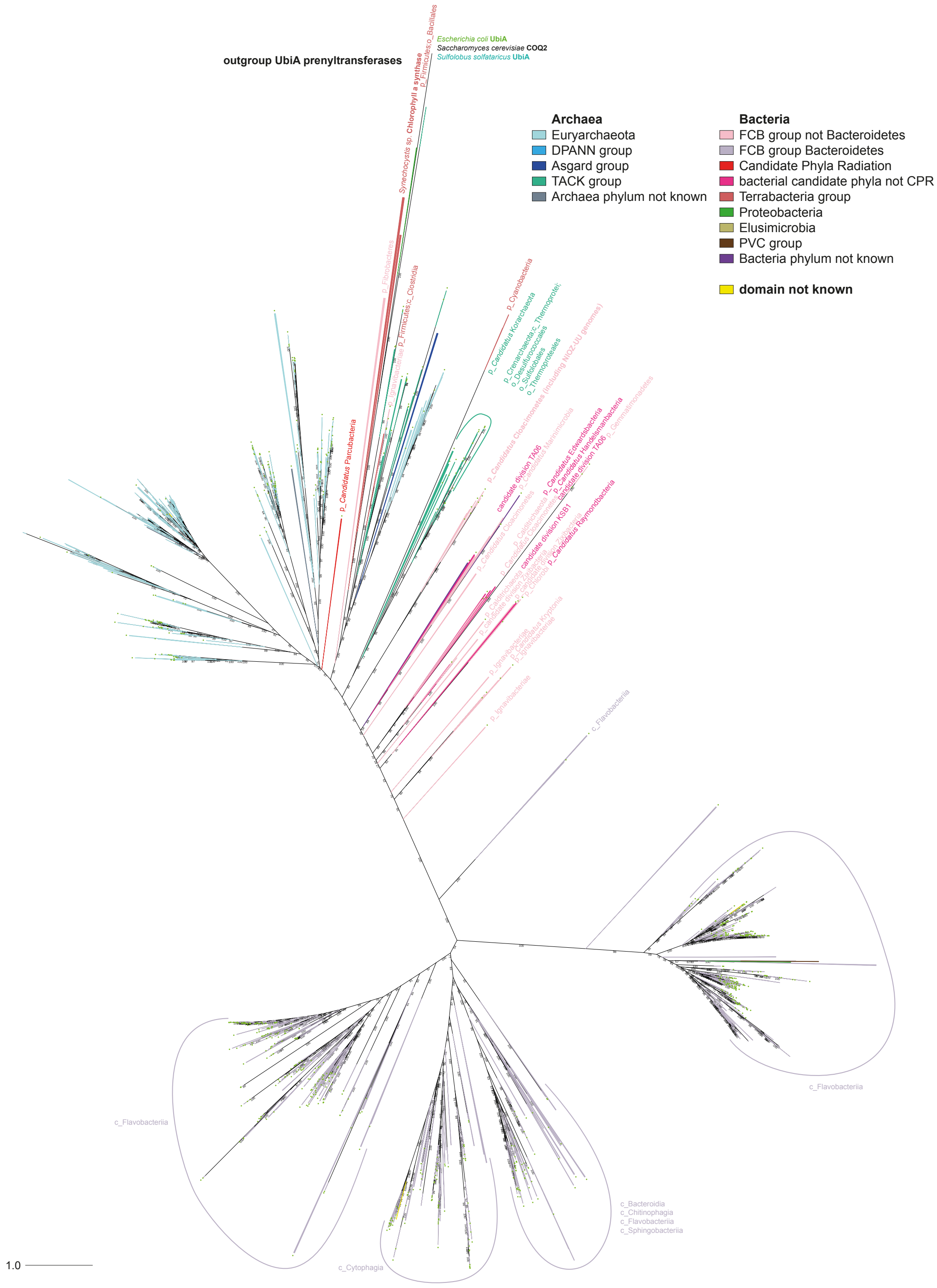

Fig. S17. Consensus tree of DGGGP synthases across the tree of life. The tree is based on 1 258 unique sequences representing 1 385 genomes from cultures and environmental samples. Bacterial clades are labeled as a certain group if a large fraction of sequences in the clade shows a consistent annotation. Interesting or aberrant placements are labeled as well, as are close archaeal sister groups to the bacterial part of the tree. Branch support is based on 1 000 ultrafast bootstraps. Scale bar represents mean number of substitutions per site. Archaeal or bacterial 'phylum not known': phylum is not known but the genome is annotated on a lower level, or sequence represents multiple groups. 'Domain not known': genomes for which no lineage was found on the PATRIC servers. Green dots: GGGP synthase is also found in the same genome, or in at least one of the genomes if the branch represents multiple genomes.

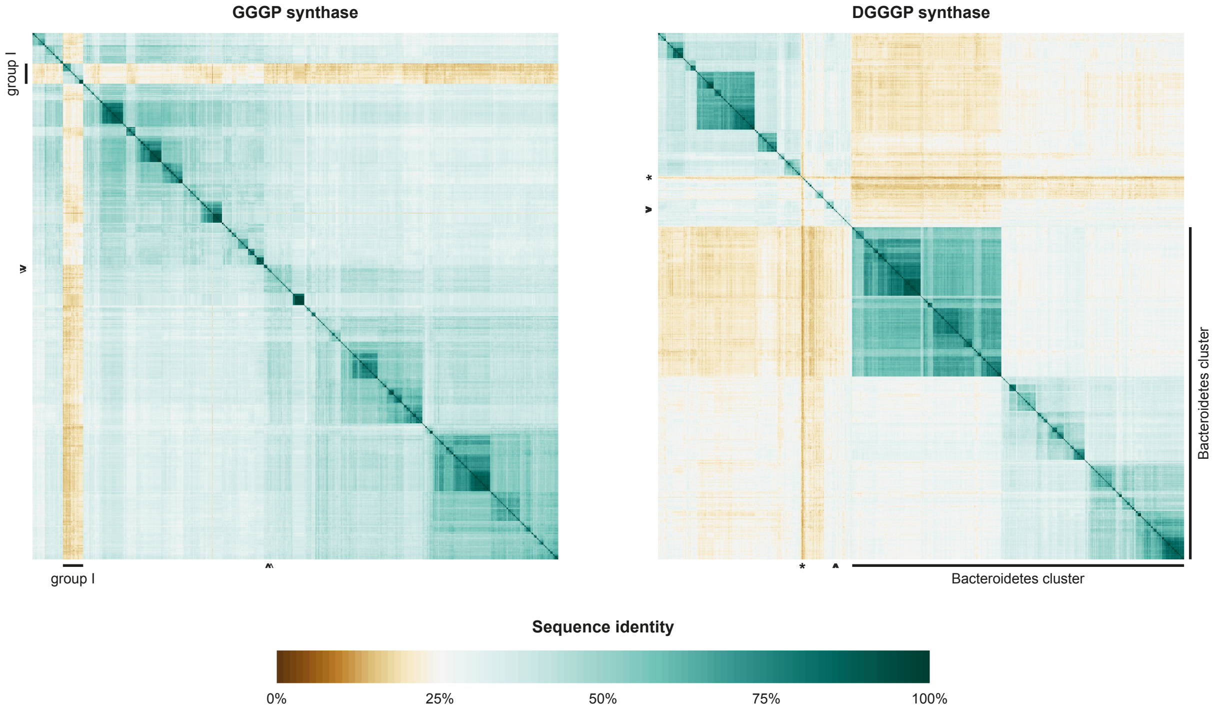

Fig. S18. Pairwise sequence identity between protein sequences used in trees for GGGP synthase and DGGGP synthase. Sequence identity was calculated excluding regions where the beginning or end of one of the sequences consisted of gaps and excluding gap-gap alignments. Sequences are ordered according to placement in the tree (Fig. S16 and Fig. S17), starting from the upper left branch. GGGP synthase sequences falling within Group I *sensu* Peterhoff et al. (18) are marked, as are DGGGP synthase sequences falling within the Bacteroidetes cluster. Asterisk indicates the position of outgroup UbiA prenyltransferases. Carets indicate the position of the enzymes from the four *Ca.* Cloacimonetes MAGs described in this study.

References Supplementary Information

1. Sollai M, Villanueva L, Hopmans EC, Reichart G-J, Sinninghe Damsté JS. A combined lipidomic and 16S rRNA gene amplicon sequencing approach reveals archaeal sources of intact polar lipids in the stratified Black Sea water column. Geobiology. 2019 Jan;17(1):91–109.

2. Moore EK, Villanueva L, Hopmans EC, Rijpstra WIC, Mets A, Dedysh SN, et al. Abundant Trimethylornithine Lipids and Specific Gene Sequences Are Indicative of Planctomycete Importance at the Oxic/Anoxic Interface in *Sphagnum*-Dominated Northern Wetlands. Appl Environ Microb. 2015 Sep;81(18):6333–44.

3. Besseling MA, Hopmans EC, Boschman RC, Sinninghe Damsté JS, Villanueva L. Benthic archaea as potential sources of tetraether membrane lipids in sediments across an oxygen minimum zone. Biogeosciences. 2018 Jul 4;15(13):4047–64.

4. Grinsven S van, Sinninghe Damsté JS, Asbun AA, Engelmann JC, Harrison J, Villanueva L. Methane oxidation in anoxic lake water stimulated by nitrate and sulfate addition. Environ Microbiol. 2019;22(2):766–82.

5. Salcher MM, Pernthaler J, Posch T. Seasonal bloom dynamics and ecophysiology of the freshwater sister clade of SAR11 bacteria “that rule the waves” (LD12). Isme J. 2011;5(8):1242–52.

6. Quast C, Pruesse E, Yilmaz P, Gerken J, Schweer T, Yarza P, et al. The SILVA ribosomal RNA gene database project: improved data processing and web-based tools. Nucleic Acids Res. 2013 Jan;41(D1):D590–6.

7. Tamura K, Stecher G, Peterson D, Filipski A, Kumar S. MEGA6: Molecular Evolutionary Genetics Analysis version 6.0. Mol Biol Evol. 2013;30(12):2725–9.

8. Dyksma S, Gallert C. *Candidatus* Syntrophosphaera thermopropionivorans: a novel player in syntrophic propionate oxidation during anaerobic digestion. Env Microbiol Rep. 2019;11(4):558–70.

9. Wang R, Lin J-Q, Liu X-M, Pang X, Zhang C-J, Yang C-L, et al. Sulfur Oxidation in the Acidophilic Autotrophic *Acidithiobacillus* spp. Front Microbiol. 2018;9:3290.

10. Wagner T, Koch J, Ermler U, Shima S. Methanogenic heterodisulfide reductase (HdrABC-MvhAGD) uses two noncubane [4Fe-4S] clusters for reduction. Science. 2017;357(6352):699–703.

11. Volkov II, Neretin LN. The Handbook of Environmental Chemistry: The Black Sea Environment. In 2008. p. 309–31.

12. Reguera G, McCarthy KD, Mehta T, Nicoll JS, Tuominen MT, Lovley DR. Extracellular electron transfer via microbial nanowires. Nature. 2005;435(7045):1098–101.

13. Stolze Y, Bremges A, Rumming M, Henke C, Maus I, Pühler A, et al. Identification and genome reconstruction of abundant distinct taxa in microbiomes from one thermophilic and three mesophilic production-scale biogas plants. Biotechnol Biofuels. 2016;9(1):156.

14. Biebl H, Pfennig N. Growth yields of green sulfur bacteria in mixed cultures with sulfur and sulfate reducing bacteria. Arch Microbiol. 1978;117(1):9–16.

15. Jannasch HW, Wirsen CO, Molyneaux SJ. Chemoautotrophic sulfur-oxidizing bacteria from the Black Sea. Deep Sea Res Part Oceanogr Res Pap. 1991;38:S1105–20.

16. Hug LA, Baker BJ, Anantharaman K, Brown CT, Probst AJ, Castelle CJ, et al. A new view of the tree of life. Nat Microbiol. 2016;1(5):16048.

17. Edgcomb VP, Taylor C, Pachiadaki MG, Honjo S, Engstrom I, Yakimov M. Comparison of Niskin vs. *in situ* approaches for analysis of gene expression in deep Mediterranean Sea water samples. Deep Sea Res Part Ii Top Stud Oceanogr. 2016;129:213–22.

18. Peterhoff D, Beer B, Rajendran C, Kumpula E, Kapetaniou E, Guldan H, et al. A comprehensive analysis of the geranylgeranylglyceryl phosphate synthase enzyme family identifies novel members and reveals mechanisms of substrate specificity and quaternary structure organization. Mol Microbiol. 2014 May;92(4):885–99.

19. Caforio A, Siliakus MF, Exterkate M, Jain S, Jumde VR, Andringa RLH, et al. Converting *Escherichia coli* into an archaebacterium with a hybrid heterochiral membrane. Proc National Acad Sci. 2018 Apr 3;115(14):3704–9.

20. Chen A, Zhang D, Poulter CD. (S)-geranylgeranylglyceryl phosphate synthase. Purification and characterization of the first pathway-specific enzyme in archaebacterial membrane lipid biosynthesis. J Biological Chem. 1993;268(29):21701–5.

21. Zhang D, Poulter CD. Biosynthesis of Archaebacterial lipids in *Halobacterium halobium* and *Methanobacterium thermoautotrophicum*. J Org Chem. 1993 Jul;58(15):3919–22.

22. Miroux B, Walker JE. Over-production of Proteins in *Escherichia coli*: Mutant Hosts that Allow Synthesis of some Membrane Proteins and Globular Proteins at High Levels. J Mol Biol. 1996 Jul 19;260(3):289–98.

23. Murakami M, Shibuya K, Nakayama T, Nishino T, Yoshimura T, Hemmi H. Geranylgeranyl reductase involved in the biosynthesis of archaeal membrane lipids in the hyperthermophilic archaeon *Archaeoglobus fulgidus*. Febs J. 2007;274(3):805–14.

24. Yokoi T, Isobe K, Yoshimura T, Hemmi H. Archaeal Phospholipid Biosynthetic Pathway Reconstructed in *Escherichia coli*. Archaea. 2012;2012(3):1–9.

25. Isobe K, Ogawa T, Hirose K, Yokoi T, Yoshimura T, Hemmi H. Geranylgeranyl Reductase and Ferredoxin from *Methanosarcina acetivorans* Are Required for the Synthesis of Fully Reduced Archaeal Membrane Lipid in *Escherichia coli* Cells. J Bacteriol. 2014 Jan;196(2):417–23.

26. Yoshinaga MY, Kellermann MY, Rossel PE, Schubotz F, Lipp JS, Hinrichs K-U. Systematic fragmentation patterns of archaeal intact polar lipids by high-performance liquid chromatography/electrospray ionization ion-trap mass spectrometry. Rapid Commun Mass Sp. 2011;25(23):3563–74.

27. Guindon S, Dufayard J-F, Lefort V, Anisimova M, Hordijk W, Gascuel O. New Algorithms and Methods to Estimate Maximum-Likelihood Phylogenies: Assessing the Performance of PhyML 3.0. Systematic Biol. 2010;59(3):307–21.

28. Abascal F, Zardoya R, Posada D. ProtTest: selection of best-fit models of protein evolution. Bioinformatics. 2005;21(9):2104–5.

29. Edgar RC. MUSCLE: a multiple sequence alignment method with reduced time and space complexity. Bmc Bioinformatics. 2004;5(1):113.

30. Castresana J. Selection of Conserved Blocks from Multiple Alignments for Their Use in Phylogenetic Analysis. Mol Biol Evol. 2000;17(4):540–52.

31. Stoddard SF, Smith BJ, Hein R, Roller BRK, Schmidt TM. rrnDB: improved tools for interpreting rRNA gene abundance in bacteria and archaea and a new foundation for future development. Nucleic Acids Res. 2014;43(Database issue):D593-8.

32. Elling FJ, Könneke M, Lipp JS, Becker KW, Gagen EJ, Hinrichs K-U. Effects of growth phase on the membrane lipid composition of the thaumarchaeon *Nitrosopumilus maritimus* and their implications for archaeal lipid distributions in the marine environment. Geochim Cosmochim Ac. 2014;141:579–97.

33. Sinninghe Damsté JS, Rijpstra WIC, Hopmans EC, Prahl FG, Wakeham SG, Schouten S. Distribution of Membrane Lipids of Planktonic Crenarchaeota in the Arabian Sea. Appl Environ Microb. 2002;68(6):2997–3002.

34. Schouten S, Pitcher A, Hopmans EC, Villanueva L, Bleijswijk J van, Sinninghe Damsté JS. Intact polar and core glycerol dibiphytanyl glycerol tetraether lipids in the Arabian Sea oxygen minimum zone: I. Selective preservation and degradation in the water column and consequences for the TEX_{86}. Geochim Cosmochim Ac. 2012;98:228–43.

35. Ghuneim L-AJ, Jones DL, Golyshin PN, Golyshina OV. Nano-Sized and Filterable Bacteria and Archaea: Biodiversity and Function. Front Microbiol. 2018;9:1971.

36. Holler T, Widdel F, Knittel K, Amann R, Kellermann MY, Hinrichs K-U, et al. Thermophilic anaerobic oxidation of methane by marine microbial consortia. Isme J. 2011;5(12):1946–56.

37. Kubo K, Lloyd KG, Biddle JF, Amann R, Teske A, Knittel K. Archaea of the Miscellaneous Crenarchaeotal Group are abundant, diverse and widespread in marine sediments. Isme J. 2012;6(10):1949–65.

38. Jahn U, Gallenberger M, Paper W, Junglas B, Eisenreich W, Stetter KO, et al. *Nanoarchaeum equitans* and *Ignicoccus hospitalis*: new insights into a unique, intimate association of two archaea. J Bacteriol. 2007;190(5):1743–50.

39. Golyshina OV, Toshchakov SV, Makarova KS, Gavrilov SN, Korzhenkov AA, Cono VL, et al. “ARMAN” archaea depend on association with euryarchaeal host in culture and in situ. Nat Commun. 2017;8(1):60.

40. Schouten S, Middelburg JJ, Hopmans EC, Sinninghe Damsté JS. Fossilization and degradation of intact polar lipids in deep subsurface sediments: A theoretical approach. Geochim Cosmochim Ac. 2010 Jul 1;74(13):3806–14.

41. Lombard J, López-García P, Moreira D. Phylogenomic Investigation of Phospholipid Synthesis in Archaea. Archaea. 2012;2012(11):1–13.

42. Liang P-H, Ko T-P, Wang AH-J. Structure, mechanism and function of prenyltransferases. Eur J Biochem. 2002;269(14):3339–54.

43. Villanueva L, Schouten S, Sinninghe Damsté JS. Phylogenomic analysis of lipid biosynthetic genes of Archaea shed light on the ‘lipid divide.’ Environ Microbiol. 2017 Jan 1;19(1):54–69.

44. Eme L, Spang A, Lombard J, Stairs CW, Ettema TJG. Archaea and the origin of eukaryotes. Nat Rev Microbiol. 2017 Nov 10;15(12):711–23.

45. Imachi H, Nobu MK, Nakahara N, Morono Y, Ogawara M, Takaki Y, et al. Isolation of an archaeon at the prokaryote-eukaryote interface. Nature. 2020;577(7791):519–25.

46. von Meijenfeldt FAB, Arkhipova K, Cambuy DD, Coutinho FH, Dutilh BE. Robust taxonomic classification of uncharted microbial sequences and bins with CAT and BAT. Genome Biol. 2019;20(1):217.

Notebook_1

July 27, 2020

[1]:
```
#/usr/bin/env python3

import matplotlib.pyplot as plt
import numpy as np
```

[2]:
```
from IPython.display import set_matplotlib_formats
set_matplotlib_formats('png', 'pdf')
```

This notebook describes the data and calculations that demonstrate that there is a striking offset between the amount of archaeal membrane lipids (intact polar lipids or IPLs) observed in the Black Sea water column and the amount of IPLs that the archaeal population can theoretically produce in the most ideal situation. We take uncertainty in measurements for both membrane lipid production and size estimates into account by taking the most extreme cases reported in literature and plotting the expected range of IPLs abundances given observed archaeal abundances. The most ideal situation, where all Archaea in the water column are at maximum known size and produce the maximum reported number of membrane lipids (i.e. have just come out of growth phase), cannot explain the observed amount of IPLs at deeper depths.

1 Functions

The following formula is used to throughout this notebook to calculate cell surface area, A, based on its radius r and length l:

$$A = 2\pi rh + 4\pi r^2$$

with

$$h = l - 2r$$

Thus, cells are modelled as spheres if $l = 2r$ (coccus shaped), and as hemisphere-capped cylinders if $l > 2r$ (rod shaped).

[3]:
```
def calc_surface_area(r, l):
    h = l - 2 * r

    return 2 * np.pi * r * h + 4 * np.pi * r ** 2
```

2 Data

In this section we define and show the data on which subsequent calculations are based.

2.1 Measured data

All measured data is a list of length 4, where each element is a different depth in the water column.

[4]:
```
depths = [500, 1000, 1500, 2000] # In m.
```

2.1.1 Archaeal lipid data

The observed archaeal lipid data is from station 4 during the BS2017 campaign and is described in Table S18. We plot it to show what it looks like.

[5]:
```
observed_IPLs = [2.52431557301726,
                 12.3592656904217,
                 14.2605958190464,
                 25.0652634330246] # In ng / L.

# Plot the data.
fig = plt.figure(figsize=(3, 5))
ax = plt.subplot2grid((1, 1), (0, 0))

ax.scatter(observed_IPLs,
           depths,
           marker='*',
           color='red',
           s=200,
           label='observed')

# Styling.
ax.set_xlim([0, 30])
ax.set_xlabel('nanograms intact polar lipids / L')
ax.set_ylim([2200, 300])
ax.set_yticks(depths)
ax.set_ylabel('depth (m)')
ax.legend(loc='upper left', bbox_to_anchor=(1, 1))
plt.suptitle('Figure 1: observed archaeal membrane lipids')

plt.show()
plt.close()
```

Figure 1: observed archaeal membrane lipids

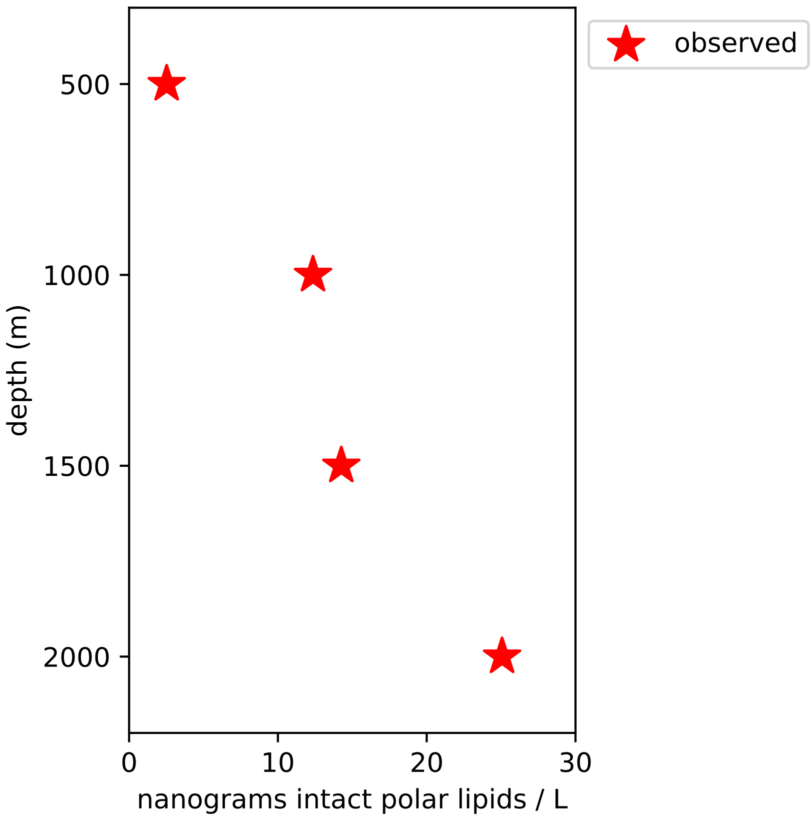

2.1.2 16S rRNA gene read data

The 16S rRNA gene read data is from station 4 during the BS2017 campaign and is described in Table S2B.

[6]:
```
total_16S = [33483253.5212389,
             35714158.946356,
             43608346.1585536,
             26792197.1985571] # In copies / L based on qPCR.

archaeal_taxa = ['Thermoplasmatales',
                 'ANME-1b',
                 'MCG+C3', # Part of the phylum Candidatus Bathyarchaeota.
                 'DHVE-6', # Part of the DPANN superphylum.
                 'Archaea, others']

# Fractions of total reads that are attributable to Archaea or
# Candidatus Cloacimonetes.
```

```
fractions = dict()
fractions['Candidatus Cloacimonetes'] = [0.0742909208685631,
                                         0.147748016535324,
                                         0.199093934608142,
                                         0.188686561035215]
fractions['Thermoplasmatales'] = [0.00221381463039215,
                                  0.00104816453435197,
                                  0.00134980791195053,
                                  0.00262319733310745]
fractions['ANME-1b'] = [0.000145926687856,
                        0.00465589190165,
                        0.0126674280968,
                        0.0157199859607]
fractions['MCG+C3'] = [0.00554165495100603,
                       0.0076276542705795,
                       0.0098257272295019,
                       0.00787755533844039]
fractions['DHVE-6'] = [0.00358054263373126,
                       0.00348603036144377,
                       0.00469427124034476,
                       0.0124533684865375]
fractions['Archaea, others'] = [0.00391510625953947,
                                0.00369801869423497,
                                0.00554131669117553,
                                0.00657199837971533]
fractions['Total Archaea'] = [sum(fractions[taxon][i] for
                                  taxon in archaeal_taxa) for
                              i, depth in enumerate(depths)]
```

We convert 16S rRNA gene read count to number of cells per liter. The following formula is used for a certain taxonomic group at a given depth, with C in cells L $^{-1}$:

$$C_{taxon} = \frac{f_{taxon}T}{n_{taxon}}$$

where f is the fraction of total reads attributable to the taxonomic group at that depth, T the total 16S count in copies L $^{-1}$ at that depth, and n the number of 16S copies per genome. We assume one 16S copy per genome for Archaea and two 16S copies per genome for *Candidatus* Cloacimonetes as "*Candidatus* Cloacimonas acidaminovorans" str. Evry has two. We plot the predicted cell counts to show what it looks like.

[7]:
```
SSU_copy_number = {taxon: 1 for
                   taxon in archaeal_taxa + ['Total Archaea']}
SSU_copy_number['Candidatus Cloacimonetes'] = 2

cells_per_L = dict()
for taxon in fractions:
    cells_per_L[taxon] = [fractions[taxon][i] * total_16S[i] /
```

```
                          SSU_copy_number[taxon] for
                          i, depth in enumerate(depths)]

# Plot the data. Only plot total archaeal abundances.
fig = plt.figure(figsize=(3, 5))
ax = plt.subplot2grid((1, 1), (0, 0))

ax.plot(cells_per_L['Total Archaea'],
        depths,
        marker='D',
        color='grey',
        linestyle='--',
        label='Total Archaea')
ax.plot(cells_per_L['Candidatus Cloacimonetes'],
        depths,
        marker='v',
        color='black',
        linestyle='--',
        label='Candidatus Cloacimonetes')

# Styling.
ax.set_xlim([0, 5000000])
ax.ticklabel_format(axis='x', style='sci', scilimits=(-2, 2))
ax.set_xlabel('cells / L')
ax.set_ylim([2200, 300])
ax.set_yticks(depths)
ax.set_ylabel('depth (m)')
ax.legend(loc='lower left', bbox_to_anchor=(1, 0))
plt.suptitle('Figure 2: observed cell counts')

plt.show()
plt.close()
```

Figure 2: observed cell counts

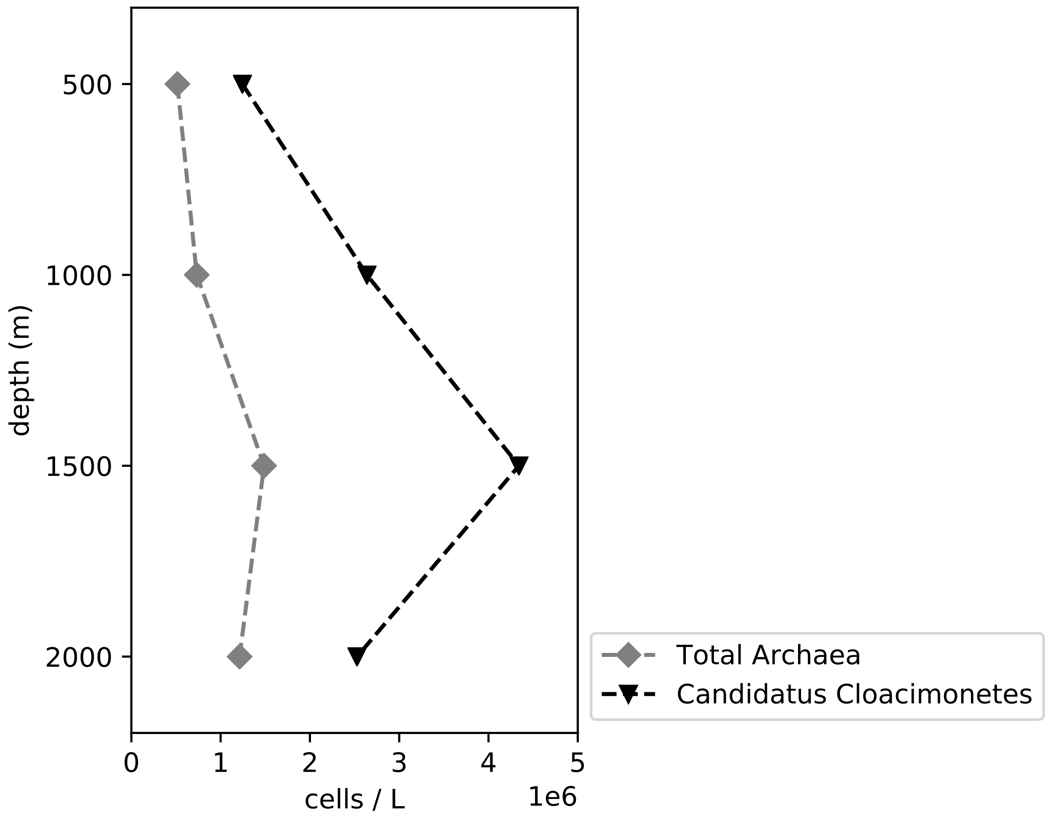

The main question this notebook addresses is: do the Archaea in the water column (Figure 2) support the observed amount of archaeal IPLs (Figure 1)?

2.2 Literature estimates for membrane lipid abundances in archaeal cells

There are three estimates for membrane lipid abundances in archaeal cells:

1. Sinninghe Damsté et al. (1) estimate the number of Crenarchaeota cells based on measured GDGT lipids. Cells are assumed rod-shaped with 0.8 μm length and a radius of 0.25 μm. Membrane lipid abundance is estimated 1.0 fg per cell.

2. Schouten et al. (2) estimate the expected amount of IPL-GDGT based on Thaumarchaeota abundance. Cells are assumed rod-shaped with 0.5 μm length and a radius of 0.075 μm. Membrane lipid abundance is estimated 0.25 fg per cell.

3. Elling et al. (3) report lipid production by marine ammonia-oxidizing Archaea in a growth experiment. Cells are assumed rod-shaped with 0.5-0.9 μm length and a radius of 0.1 μm. Membrane lipid abundance estimates range from 0.86 fg per cell for small cells in the early growth phase to 1.85 fg per cell for large cells right after growth phase. This represents an increased production of ~20% per cell surface area right after growth. Stationary phase

production is lower again at 0.92 fg per cell.

We construct the dictionary 'estimates' that contains per study both the estimated membrane lipids per cell and their estimated surfaces. Multiple estimates indicate a range. We print the dictionary to show what it looks like.

[8]:
```python
estimates = dict()
(estimates['Sinninghe Damsté'], estimates['Schouten'],
 estimates['Elling']) = dict(), dict(), dict()

# Amount of lipids per cell are given in femtogram, radius and length in
# micrometre.
estimates['Sinninghe Damsté']['lipids per cell'] = [1.0]
estimates['Sinninghe Damsté']['surface area'] = [calc_surface_area(0.25,
                                                                   0.8)]

estimates['Schouten']['lipids per cell'] = [0.25]
estimates['Schouten']['surface area'] = [calc_surface_area(0.075, 0.5)]

estimates['Elling']['lipids per cell'] = [0.86, 1.85]
estimates['Elling']['surface area'] = [calc_surface_area(0.1, l) for
                                       l in [0.5, 0.9]]

# Print. Limit precision for the display of floats to 5 decimal points.
for i, study in enumerate(estimates):
    print(study)
    print('membrane lipids per cell (fg):\t',
          [float(f'{v:.5f}') for v in
           estimates[study]['lipids per cell']])
    print('cell surface area (micrometre^2):\t',
          [float(f'{v:.5f}') for v in
           estimates[study]['surface area']])
    if i < 2:
        print()
```

```
Sinninghe Damsté
membrane lipids per cell (fg):   [1.0]
cell surface area (micrometre^2):        [1.25664]

Schouten
membrane lipids per cell (fg):   [0.25]
cell surface area (micrometre^2):        [0.23562]

Elling
membrane lipids per cell (fg):   [0.86, 1.85]
cell surface area (micrometre^2):        [0.31416, 0.56549]
```

2.3 Estimates of cell surface area of different archaeal taxa based on reported size ranges in literature

Size estimates for the four encountered archaeal taxa are:

- Thermoplasmatales: rod-shaped cells with a length of 0.5-3.0 μm and radius of 0.1-0.25 μm (4).
- ANME-1: cells within aggregates with 1.2 μm length and a radius of 0.15-0.2 μm (5).
- Candidatus Bathyarchaeota: spherical cells with a radius of 0.2-0.25 μm (6).
- DPANN: spherical cells with a radius of 0.2-0.25 μm, based on the *Nanoarchaeum equitans* (7) and ARMAN archaea (8).

We calculate the upper and lower bound of cell surface area by taking the smallest and largest combination of reported l and r. We construct the dictionary 'surface_area_range' that contains the range of surface areas for different archaeal taxa, and print it to show what it looks like.

[9]:
```
surface_area_range = dict()
# Radius and length are given in micrometre.
surface_area_range['Thermoplasmatales'] = [calc_surface_area(r, l) for
                                           r in [0.1, 0.25] for
                                           l in [0.5, 3.0]]
surface_area_range['ANME-1'] = [calc_surface_area(r, 1.2) for
                                r in [0.15, 0.2]]
surface_area_range['Bathyarchaeota'] = [calc_surface_area(r, 2 * r) for
                                        r in [0.2, 0.25]]
surface_area_range['DPANN'] = [calc_surface_area(r, 2 * r) for
                               r in [0.2, 0.25]]

# Print. Limit precision for the display of floats to 5 decimal points.
print('range of cell surface area (micrometre^2)\n')
for i, taxon in enumerate(sorted(surface_area_range)):
    print('{0}:\t{1}'.format(taxon,
                              [float(f'{v:.5f}') for v in
                               surface_area_range[taxon]]))
```

```
range of cell surface area (micrometre^2)

ANME-1: [1.13097, 1.50796]
Bathyarchaeota: [0.50265, 0.7854]
DPANN:  [0.50265, 0.7854]
Thermoplasmatales:      [0.31416, 1.88496, 0.7854, 4.71239]
```

3 Calculations

Next, we calculate the theoretical range of membrane lipids that the Archaea in the water column can produce.

3.1 Predictions of membrane lipids per cell for different archaeal taxa

We predict how many lipids per cell the different archaeal taxa produce, based on membrane lipid abundance estimates by Sinninghe Damsté et al., Schouten et al., and Elling et al., and their membrane surface area relative to the surface area of the organisms investigated in the respective studies. We estimate the number of lipids per cell, L, with the formula:

$$L_{taxon} = \frac{A_{taxon} L_{study}}{A_{study}}$$

where L_{study} and A_{study} are the membrane lipid estimate and surface area of the organisms in that particular study, respectively. We calculate L_{taxon} for the upper and lower surface area bounds of that taxon, and for the upper and lower bounds of cell surface area and membrane lipid abundance estimates reported in the particular study. The upper and lower bounds of all these combinations are reported. For the 'other Archaea', we assume a cell membrane lipid abundance between 0.25 and 5 femtogram per cell. We print the dictionary 'lipids_per_cell' to show what it looks like.

[10]:
```
def predict_lipids_per_cell(surface_areas, estimates, study):
    """surface areas = list of cell surface areas.
       estimates = the estimates dictionary.
       study = either Sinninghe Damsté, Schouten, or Elling.
       """
    lipids = list()

    for surface_area in surface_areas:
        lipids.append(surface_area *
                      min(estimates[study]['lipids per cell']) /
                      min(estimates[study]['surface area']))
        lipids.append(surface_area *
                      max(estimates[study]['lipids per cell']) /
                      max(estimates[study]['surface area']))

    return (min(lipids), max(lipids))
```

[11]:
```
# Amount of lipids per cell are given in femtogram.
lipids_per_cell = dict()
for study in estimates:
    lipids_per_cell[study] = dict()
    lipids_per_cell[study]['ANME-1b'] = predict_lipids_per_cell(
        surface_area_range['ANME-1'],
        estimates,
        study)
    lipids_per_cell[study]['DHVE-6'] = predict_lipids_per_cell(
        surface_area_range['DPANN'],
        estimates,
        study)
    lipids_per_cell[study]['MCG+C3'] = predict_lipids_per_cell(
        surface_area_range['Bathyarchaeota'],
```

```
        estimates,
        study)
    lipids_per_cell[study]['Thermoplasmatales'] = predict_lipids_per_cell(
        surface_area_range['Thermoplasmatales'],
        estimates,
        study)
    lipids_per_cell[study]['Archaea, others'] = (0.25, 5.0)

# Print. Limit precision for the display of floats to 5 decimal points.
print('minimum and maximum membrane lipid abundance estimates (fg / cell)\n')
for i, study in enumerate(lipids_per_cell):
    print(study)
    for taxon in lipids_per_cell[study]:
        print('{0}:\t{1}'.format(taxon,
                                 tuple([float(f'{v:.5f}') for v in
                                        lipids_per_cell[study][taxon]])))
    if i < 2:
        print()
```

```
minimum and maximum membrane lipid abundance estimates (fg / cell)

Sinninghe Damsté
ANME-1b:        (0.9, 1.2)
DHVE-6: (0.4, 0.625)
MCG+C3: (0.4, 0.625)
Thermoplasmatales:      (0.25, 3.75)
Archaea, others:        (0.25, 5.0)

Schouten
ANME-1b:        (1.2, 1.6)
DHVE-6: (0.53333, 0.83333)
MCG+C3: (0.53333, 0.83333)
Thermoplasmatales:      (0.33333, 5.0)
Archaea, others:        (0.25, 5.0)

Elling
ANME-1b:        (3.096, 4.93333)
DHVE-6: (1.376, 2.56944)
MCG+C3: (1.376, 2.56944)
Thermoplasmatales:      (0.86, 15.41667)
Archaea, others:        (0.25, 5.0)
```

3.2 Calculate the range of amount of lipid molecules produced by the Archaea in the water column

The amount of lipid molecules produced by an archaeal taxon in the water column, M is calculated as:

$$M_{taxon} = C_{taxon} L_{taxon}$$

We calculate minimum and maximum values based on the possible ranges of estimates, and sum the values for all archaeal taxa.

[12]:
```
predictions_low = dict()
predictions_high = dict()

for study in lipids_per_cell:
    predictions_low[study] = [0 for depth in depths]
    predictions_high[study] = [0 for depth in depths]

    for i, depth in enumerate(depths):
        for taxon in archaeal_taxa:
            predictions_low[study][i] += (
                cells_per_L[taxon][i] *
                min(lipids_per_cell[study][taxon]) /
                1000000) # Convert fg to ng.
            predictions_high[study][i] += (
                cells_per_L[taxon][i] *
                max(lipids_per_cell[study][taxon]) /
                1000000) # Convert fg to ng.
```

The expected ranges of IPLs the archaeal population in the water column can theoretically support differs per study and looks like this:

[13]:
```
# Plot the data.
fig = plt.figure(figsize=(3, 5))
ax = plt.subplot2grid((1, 1), (0, 0))

ax.fill_betweenx(depths,
                 predictions_low['Elling'],
                 predictions_high['Elling'],
                 color='red',
                 alpha=0.25,
                 linewidth=0,
                 label='Elling et al.')
ax.fill_betweenx(depths,
                 predictions_low['Schouten'],
                 predictions_high['Schouten'],
                 color='blue',
                 alpha=0.25,
                 linewidth=0,
                 label='Schouten et al.')
ax.fill_betweenx(depths,
                 predictions_low['Sinninghe Damsté'],
                 predictions_high['Sinninghe Damsté'],
```

```
                 color='green',
                 alpha=0.25,
                 linewidth=0,
                 label='Sinninghe Damsté et al.')

# Styling.
ax.set_xlim([0, 7])
ax.set_xlabel('nanograms intact polar lipids / L')
ax.set_ylim([2200, 300])
ax.set_yticks(depths)
ax.set_ylabel('depth (m)')
ax.legend(loc='upper left', bbox_to_anchor=(1, 1))
plt.suptitle('Figure 3: expected archaeal membrane lipids')

plt.show()
plt.close()
```

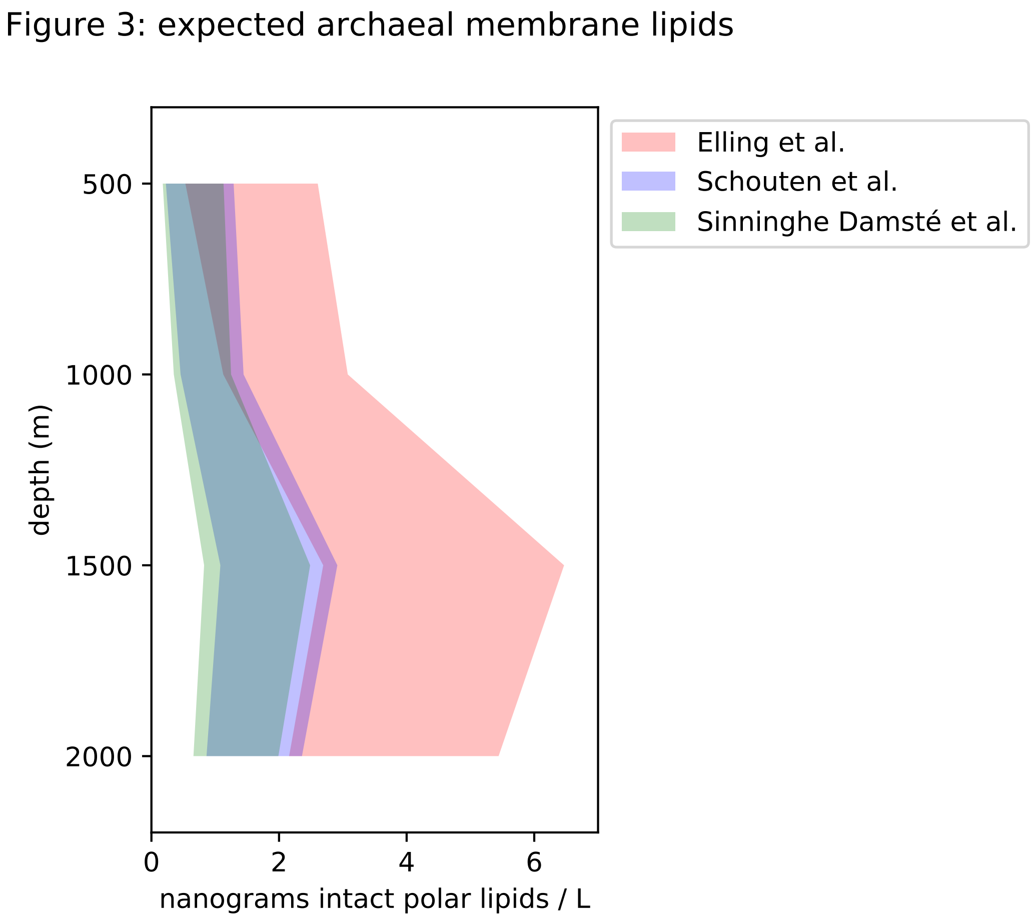

The theoretical maximum of expected IPLs in the water column, i.e. the maximum predictions

based on Elling et al., represents an ideal situation:

- All Archaea in the water column should be the maximum size reported in literature.
- All Archaea in the water column should produce the maximum reported amount of lipids per cell surface area. In Elling et al. this only happens right after growth phase, in the early stationary phase.

Both of these conditions are unlikely in the deep waters of the Black Sea. Nevertheless, even if we assume both, there is still an offset between expected and observed IPLs in the water column, which can be seen if we plot expected and observed together.

4 Plot expected versus observed amount of archaeal lipids in the water column, together with abundances of Archaea and *Candidatus* Cloacimonetes

[14]:
```
# Plot the data.
fig = plt.figure(figsize=(3.5, 7))
ax1 = plt.subplot2grid((1, 1), (0, 0))

# Plot abundances of Archaea and Candidatus Cloacimonetes.
ax1.plot(cells_per_L['Total Archaea'],
         depths,
         marker='D',
         color='grey',
         linestyle='--',
         label='Total Archaea')
ax1.plot(cells_per_L['Candidatus Cloacimonetes'],
         depths,
         marker='v',
         color='black',
         linestyle='--',
         label='Candidatus Cloacimonetes')

# Styling.
ax1.set_xlim([0, 8e6])
ax1.ticklabel_format(axis='x', style='sci', scilimits=(-2, 2))
ax1.set_xlabel('cells / L')
ax1.set_ylim([2200, 300])
ax1.set_yticks(depths)
ax1.set_ylabel('depth (m)')
ax1.legend(loc='lower left', bbox_to_anchor=(1, 0))

ax2 = ax1.twiny()

# Plot predicted and observed membrane lipids.
ax2.fill_betweenx(depths,
                  predictions_low['Elling'],
```

```
                  predictions_high['Elling'],
                  color='red',
                  alpha=0.25,
                  linewidth=0,
                  label='Elling et al.')
ax2.fill_betweenx(depths,
                  predictions_low['Schouten'],
                  predictions_high['Schouten'],
                  color='blue',
                  alpha=0.25,
                  linewidth=0,
                  label='Schouten et al.')
ax2.fill_betweenx(depths,
                  predictions_low['Sinninghe Damsté'],
                  predictions_high['Sinninghe Damsté'],
                  color='green',
                  alpha=0.25,
                  linewidth=0,
                  label='Sinninghe Damsté et al.')
ax2.scatter(observed_IPLs,
            depths,
            marker='*',
            color='red',
            s=200,
            label='observed')

# Styling.
ax2.set_xlim([0, 30])
ax2.set_xlabel('nanograms archaeal intact polar lipids / L')
ax2.legend(loc='upper left', bbox_to_anchor=(1, 1))
plt.suptitle('Figure 4: observed versus expected archaeal membrane lipids')

plt.show()
plt.close()
```

Figure 4: observed versus expected archaeal membrane lipids

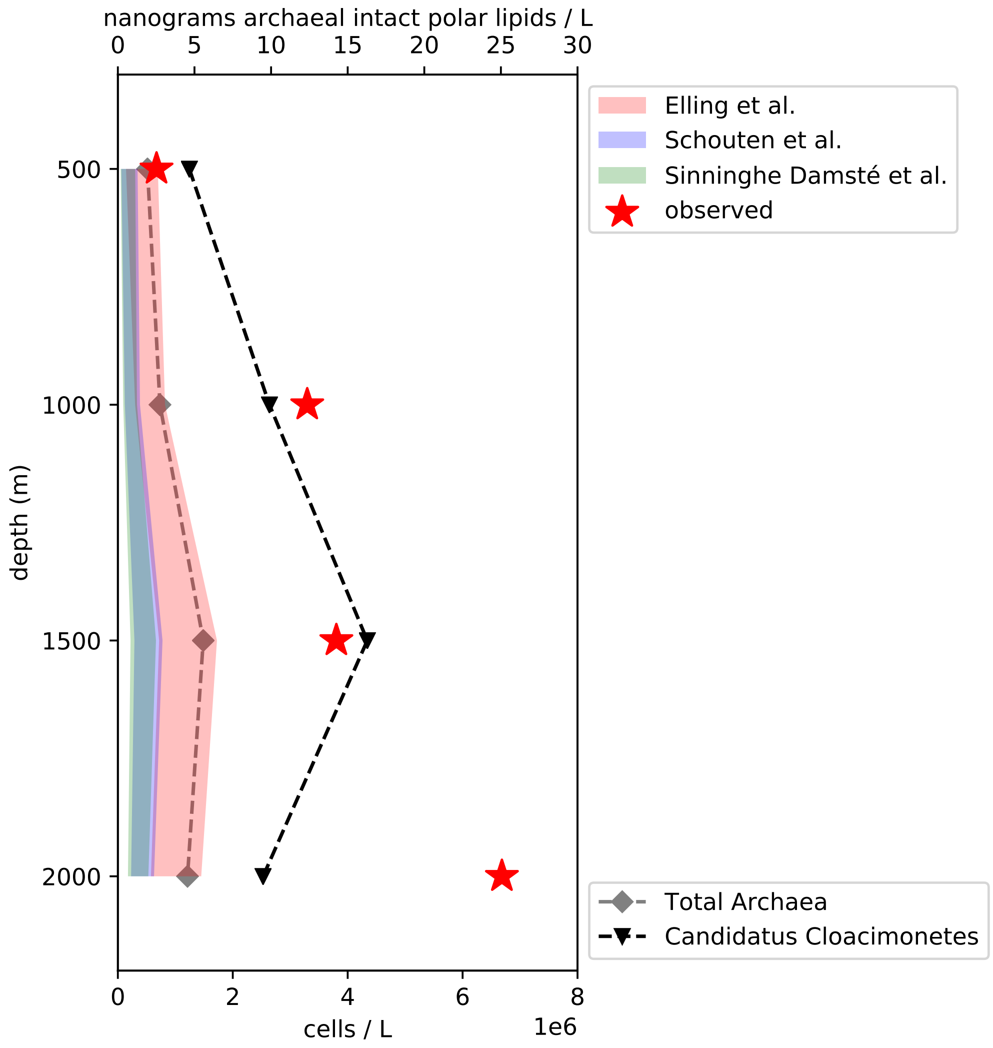

Thus, at 1000m, 1500m, and 2000m there is a clear offset between observed IPLs and expected IPLs based on archaeal abundances in the water column.

---

5 References

1. Sinninghe Damsté JS, Rijpstra WIC, Hopmans EC, Prahl FG, Wakeham SG, Schouten S. Distribution of Membrane Lipids of Planktonic Crenarchaeota in the Arabian Sea. Appl Environ Microb. 2002;68(6):2997–3002.

2. Schouten S, Pitcher A, Hopmans EC, Villanueva L, Bleijswijk J van, Sinninghe Damsté JS. Intact polar and core glycerol dibiphytanyl glycerol tetraether lipids in the Arabian Sea oxygen minimum zone: I. Selective preservation and degradation in the water column and

consequences for the TEX86. Geochim Cosmochim Ac. 2012;98:228–43.

3. Elling FJ, Könneke M, Lipp JS, Becker KW, Gagen EJ, Hinrichs K-U. Effects of growth phase on the membrane lipid composition of the thaumarchaeon *Nitrosopumilus maritimus* and their implications for archaeal lipid distributions in the marine environment. Geochim Cosmochim Ac. 2014;141:579–97.

4. Ghuneim L-AJ, Jones DL, Golyshin PN, Golyshina OV. Nano-Sized and Filterable Bacteria and Archaea: Biodiversity and Function. Front Microbiol. 2018;9:1971.

5. Holler T, Widdel F, Knittel K, Amann R, Kellermann MY, Hinrichs K-U, et al. Thermophilic anaerobic oxidation of methane by marine microbial consortia. Isme J. 2011;5(12):1946–56.

6. Kubo K, Lloyd KG, Biddle JF, Amann R, Teske A, Knittel K. Archaea of the Miscellaneous Crenarchaeotal Group are abundant, diverse and widespread in marine sediments. Isme J. 2012;6(10):1949–65.

7. Jahn U, Gallenberger M, Paper W, Junglas B, Eisenreich W, Stetter KO, et al. *Nanoarchaeum equitans* and *Ignicoccus hospitalis*: new insights into a unique, intimate association of two archaea. J Bacteriol. 2007;190(5):1743–50.

8. Golyshina OV, Toshchakov SV, Makarova KS, Gavrilov SN, Korzhenkov AA, Cono VL, et al. “ARMAN” archaea depend on association with euryarchaeal host in culture and in situ. Nat Commun. 2017;8(1):60.
